# APMAP interacts with lysyl oxidase–like proteins, and disruption of *Apmap* leads to beneficial visceral adipose tissue expansion

**DOI:** 10.1096/fj.201601337R

**Published:** 2017-05-30

**Authors:** Ariane R. Pessentheiner, Katharina Huber, Helmut J. Pelzmann, Andreas Prokesch, Franz P. W. Radner, Heimo Wolinski, Josefine Lindroos-Christensen, Gerald Hoefler, Thomas Rülicke, Ruth Birner-Gruenberger, Martin Bilban, Juliane G. Bogner-Strauss

**Affiliations:** * Institute of Biochemistry, Graz University of Technology, Graz, Austria;; † Institute of Cell Biology, Histology, and Embryology, Medical University of Graz, Graz, Austria;; ‡ Institute of Molecular Biosciences, University of Graz, Graz, Austria;; § BioTechMed-Graz, University of Graz, Graz, Austria;; ¶ Department of Laboratory Medicine, Medical University of Vienna, Vienna, Austria;; ‖ Institute of Pathology, Medical University of Graz, Graz, Austria;; # Institute of Laboratory Animal Science, University of Veterinary Medicine Vienna, Vienna, Austria;; ** Omics Center Graz, BioTechMed-Graz, University of Graz, Graz, Austria

**Keywords:** obesity, extracellular matrix, insulin resistance

## Abstract

Adipocyte plasma membrane–associated protein (APMAP) has been described as an adipogenic factor in 3T3-L1 cells with unknown biochemical function; we therefore aimed to investigate the physiologic function of APMAP *in vivo*. We generated Apmap*-*knockout mice and challenged them with an obesogenic diet to investigate their metabolic phenotype. We identified a novel truncated adipocyte-specific isoform of APMAP in mice that is produced by alternative transcription. Mice lacking the full-length APMAP protein, the only isoform that is expressed in humans, have an improved metabolic phenotype upon diet-induced obesity, indicated by enhanced insulin sensitivity, preserved glucose tolerance, increased respiratory exchange ratio, decreased inflammatory marker gene expression, and reduced adipocyte size. At the molecular level, APMAP interacts with the extracellular collagen cross-linking matrix proteins lysyl oxidase–like 1 and 3. On a high-fat diet, the expression of lysyl oxidase–like 1 and 3 is strongly decreased in Apmap-knockout mice, paralleled by reduced expression of profibrotic collagens and total collagen content in epididymal white adipose tissue, indicating decreased fibrotic potential. Together, our data suggest that APMAP is a novel regulator of extracellular matrix components, and establish that APMAP is a potential target to mitigate obesity-associated insulin resistance.—Pessentheiner, A. R., Huber, K., Pelzmann, H. J., Prokesch, A., Radner, F. P. W., Wolinski, H., Lindroos-Christensen, J., Hoefler, G., Rülicke, T., Birner-Gruenberger, R., Bilban, M., Bogner-Strauss, J. G. APMAP interacts with lysyl oxidase–like proteins, and disruption of *Apmap* leads to beneficial visceral adipose tissue expansion.

Adipose tissue (AT) is the biggest caloric reservoir of the body and plays a central role in regulating whole-body energy metabolism (1). During sufficient nutrient supply, excessive energy is stored in adipocytes in the form of lipid droplets. This energy is accessible during periods of nutrient deprivation, such as fasting or physical activity (2). When the caloric intake constantly exceeds energy expenditure, AT expands and people become obese. Progression of obesity decreases the metabolic flexibility and functionality of adipocytes, accompanied by increased inflammation, ectopic fat accumulation, and systemic insulin resistance, which ultimately leads to type 2 diabetes. Although the notion of healthy adiposity was recently challenged (3), a significant proportion of obese people are considered to be metabolically healthy, without major obesity-attributed adverse effects (4, 5), suggesting the concept of healthy AT expansion. AT expansion is achieved either by elevating adipocyte numbers (hyperplasia) or by increasing the size of already-existing adipocytes (hypertrophy). Adipocytes are surrounded by an extracellular matrix (ECM) that provides structural and mechanical support and is involved in various signaling events (6).

During AT expansion, dynamic remodeling of the ECM is crucial because imbalances in ECM synthesis and degradation lead to fibrosis. Fibrosis is a hallmark of AT dysfunction, which is strongly associated with inflammatory processes and the progression of insulin resistance (7–9). In adipocytes, the ECM undergoes structural changes during differentiation from a fibrillar to a laminar structure (10–12). The fibrillar structure of preadipocytes, mainly containing collagen I, plasmin, and fibronectin, is replaced by a laminar structure built by collagen VI, laminin, and a high amount of collagen IV (10, 11). Lysyl oxidase (LOX) and lysyl oxidase-like 1–4 (LOXL1–4) proteins mediate the cross-linking of collagens and/or elastin and are thus relevant in the remodeling of the ECM (13). Only LOX (9, 14) and LOXL1 (15) have been described to be expressed in AT. LOX is a secreted protein that is down-regulated within the first phase of adipocyte differentiation (9, 16). In obese AT, LOX expression is up-regulated in a hypoxia-dependent manner and is implicated in tissue fibrosis (9). Inhibition of systemic LOX activity with β-aminopropionitrile reduced body weight and improved the metabolic profile in obese rats (14). However, nothing is known about other lysyl oxidase family members in the context of AT ECM.

In this study, we concentrated on the physiologic characterization of adipocyte plasma membrane–associated protein (APMAP) in the context of obesity. We and others (17, 18) showed that *Apmap* expression is highly up-regulated during adipogenic differentiation of various murine and human cell lines. Further, we showed that *Apmap* expression is important for adipogenesis *in vitro* (17). APMAP is a 46 kDa glycosylated type II transmembrane protein with an N-terminal anchor and a 6-bladed β-propeller extracellular domain with potential hydrolase activity and calcium binding (17). Because the C-terminal region of APMAP faces the extracellular space, a function in regulating ECM is conceivable.

Here we identified LOXL1 and -3 as interaction partners of APMAP. When fed a high-fat diet (HFD), Apmap exon 1–knockout (ApmapE1-KO) mice show a strongly reduced expression of these LOXLs in the epididymal white adipose tissue (eWAT) with a concurrent reduction of collagen content. Furthermore, ApmapE1-KO mice show a decreased adipocyte size in eWAT and an improved metabolic phenotype upon diet-induced obesity (DIO). Interestingly, in mice a second AT-specific APMAP protein is expressed that does not exist in humans; however, our mouse model lacks the exact full-length APMAP protein version (APMAP_E1) that is conserved in humans. These data suggest a novel function for APMAP in ECM remodeling, thereby linking the properties of ECM to the development of insulin resistance.

## MATERIALS AND METHODS

### Cell culture

3T3-L1 and COS7 [American Type Culture Collection (ATCC), Manassas, VA, USA] cells were grown in DMEM (4.5 g/L glucose) supplemented with 10% fetal bovine serum (FBS), l-glutamine, and penicillin–streptomycin at 37°C and 5% CO_2_. Differentiation of 3T3-L1 cells and stable silencing of *Apmap* in 3T3-L1 cells have been previously described in detail. Stable overexpression of Apmap in 3T3-L1 cells was achieved as previously described by us (17).

### Animal studies

We flanked exon 1 of the *Apmap* gene with 2 loxP sites and cloned the homologous regions into a targeting vector that was electroporated into 129 HM-1 embryonic stem (ES) cells. Homologous and Cre recombined ES cells haboring the floxed allele were injected into C57BL/6 blastocysts, and chimeric males were tested for germ-line transmission. Heterozygous floxed mice were bred with CMV-Cre mice (19) to gain heterozygous KO mice. Mice were backcrossed to the C57BL/6J background for at least 7 generations. Homozygous ApmapE1-KO mice were fertile and were used for breeding. If not otherwise stated, male ApmapE1-KO and wild-type (WT) mice were used for this study. Animal age is mentioned in figures and text. Mice were housed in groups of 2–5 in filter-top cages in a pathogen-free barrier facility. Mice had free access to food and water and were maintained in a 13-h light/11-h dark cycle. They were either fed a normal rodent chow diet (Ssniff, Soest, Germany; 11% calories from fat, 53% carbohydrates, and 36% protein) or received an HFD (Ssniff; 45% calories from fat, 35% carbohydrates, and 20% protein) at the age of 8–9 wk. The number of mice used for each experiment is stated in the figures. During the study, the mice were weighed weekly and food intake was monitored. Tissues were collected from mice and snap-frozen in liquid nitrogen. Animal experiments were approved and carried out according to the guidelines of the Austrian Federal Ministry of Science and Research, Division of Genetic Engineering and Animal Experiments.

### Total RNA extraction and cDNA synthesis

Total RNA was isolated from cells using the peqGold Total RNA Kit (Peqlab Biotechnologie, Erlangen, Germany) and from tissue with Trizol reagent (Thermo Fisher Scientific, Waltham, MA, USA) according to the manufacturer’s protocols. cDNA was generated using the QuantiTect Reverse Transcription kit (Qiagen, Germantown, MD, USA). mRNA expression was assessed using the StepOne Plus Detector system and SYBR Green PCR master mix (Thermo Fisher Scientific). Gene expression was normalized to TfIIβ. Relative mRNA expression levels were calculated using averaged ΔΔ*C_t_* values for each biologic replicate (20). Primers are listed in **Table 1**.

**TABLE 1.** Murine primer sequences used for qPCR

	Primer, 5′−3′	
Target gene	Forward	Reverse
*Apmap E7/8*	GAAGACTTTGTCCTAGTGGCAG	ATTGTCAGGAAATCCAGGCATG
*Apmap_E1*	GAGTGTCAAGGCGCTGTTTGG	GGCCATCGTCCGTGACGAC
*Apmap_E2*	GCTTGTCAGTGTGTGTGGCTC	CTGAAACTCTGAGGATCTATGG
*Apmap_E3*	GTTGAACTTGGGCTGGTTATAG	TTCTGCTTGCCGCAACTTCG
*Apmap_E6*	CATGGTGACTGTTTAGGGAGG	TAGGTTCATCATCTCGGGTTTC
*Cebpα*	ATCTGCGAGCACGAGACGTC	TGTCGGCTGTGCTGGAAGA
*Cidea*	TGACATTCATGGGATTGCAGAC	GGCCAGTTGTGATGACTAAGAC
*Col1a1*	ATGGATTCCCGTTCGAGTACG	TCGGTGGACATTAGGCGC
*Col3a1*	CTCTTATTTTGGCACAGCAGTC	ATTTGACATGGTTCTGGCTTCC
*Col4a1*	GTGTGCATGAGAAGAACATAAC	TTCTAGGGTTCATTGCTGTTAC
*Col6a1*	CACACATACCGGCGCAATT	TCTGGCAGCCTGGCACTC
*Col6a2*	ATCGCTAACTCTCCACATGAGCTC	AGCTCACCTTGTAGCACTCTCCA
*Col6a3*	ATCAACCTCATGGTGAACACAG	TCTCTAGGTCATAGTGCCATAG
*Emr1*	CTTTGGCTATGGGCTTCCAGTC	GCAAGGAGGACAGAGTTTATCGTG
*Il-1b*	GAAATGCCACCTTTTGACAGTG	TGGATGCTCTCATCAGGACAG
*Il-10*	GCTGGACAACATACTGCTAACC	GCATCACTTCTACCAGGTAAAAC
*Il-6*	CCAGAGTCCTTCAGAGAGATAC	CTTATCTGTTAGGAGAGCATTGG
*Lox*	AGCTGTCACCAACATTACCAC	AGCTTGCTTTGTGGCCTTCA
*Loxl1*	ACGTGCAGAGAGCCCATCTG	GGGAAGCGCAATAGCACTCG
*Loxl3*	TGATGATGACTTCACGCTGCAG	AGATTGTCCAACCAGATTCGGC
*Mcp1*	TTAAAAACCTGGATCGGAACCAA	GCATTAGCTTCAGATTTACGGGT
*Plin1*	GGTACACTATGTGCCGCTTCC	CTTTGCGCTCCGCCTCT
*Pparγ2*	TGCCTATGAGCACTTCACAAGAAAT	CGAAGTTGGTGGGCCAGAA
*Prdm16*	TCCACAGCACGGTGAAGCCA	ATCTGCGTCCTGCAGTCGGC
*TFIIβ*	GTCACATGTCCGAATCATCCA	TCAATAACTCGGTCCCCTACAA
*Tgfβ*	CTCCCGTGGCTTCTAGTGC	GCCTTAGTTTGGACAGGATCTG
*Tnfα*	ATTCGAGTGACAAGCCTGTAGC	GGTTGTCTTTGAGATCCATGCC
*Ucp1*	ACACCTGCCTCTCTCGGAAA	TAGGCTGCCCAATGAACACT
*Atf4*	GTTGGTCAGTGCCTCAGACA	CATTCGAAACAGAGCATCG
*Atf6*	TTATCAGCATACAGCCTGCG	CTTGGGACTTTGAGCCTCTG
*Ire1*	CCCTGATAGGTTGAATCCTGGCTATGTG	AATCTATGCGCTAATCTGCT3GGCCTCTG
*Xbp1s*	GAGTCCGCAGCAGGTG	GTGTCAGAGTCCATGGGA
*Chop10*	CTGCCTTTCACCTTGGAGAC	CGTTTCCTGGGGATGAGATA
*Bax*	TCCAGACAAGCAGCCGCTCA	TGCTGACGTGGACACGGACT
*Pgc1-a*	TCTCTGGAACTGCAGGCCTAAC	TCAGCTTTGGCGAAGCCTT
*Ucp2*	GTTCCTCTGTCTCGTCTTGC	GGCCTTGAAACCAACCA
*Ucp3*	AAGGATTTGTGCCCTCCTTTCT	AAAACGGAGATTCCCGCA
*Ppara*	CCTGAACATCGAGTGTCGAATATG	GCGAATTGCATTGTGTGACATC
*Nrf1*	GAAACGGAAACGGCCTCATG	ACTCGCGTCGTGTACTCATC
*Tfam*	TCCCCTCGTCTATCAGTCTTG	AATTTGGGTAGCTGTTCTGTGG
*Nox2*	CATCGGTGACAATGAGAACG	AAGGCCGATGAAGAAGATCA
*Nox4*	ATCTTTGCCTCGAGGGTTTT	TGACAGGTTTGTTGCTCCTG
*Ncf1*	TCCCTGCATCCTATCTGGAG	TCCAGGAGCTTATGAATGACC
*Nos2*	GTTCTCAGCCCAACAATACAAGA	GTGGACGGGTCGATGTCAC

### Northern blot analysis

Total RNA (10 µg) was resolved by formaldehyde/agarose gel electrophoresis and blotted onto a Hybond-N^+^ membrane (GE Healthcare, Waukesha, WI, USA). The mRNA was hybridized with a murine Apmap cDNA probe generated by PCR (primer located in the middle 5′-CCAGGATCCTGTTGGACCAGTTGCAGTTC-3′ and at the 3′ end of the Apmap coding sequence) and labeled with [α-[^32^P]]dATP (GE Healthcare) using the Prime-a-Gene DNA labeling kit according to the manufacturer’s protocol (Promega, Madison, WI, USA). After hybridization and washing, signals were visualized by exposure to a PhosphorImager Screen (GE Healthcare). Transcripts discussed in the results section were obtained from GenBank [National Center for Biotechnology Information (NCBI), Bethesda, MD, USA; *https://www.ncbi.nlm.nih.gov/genbank/*) and The European Bioinformatics Institute (Ensembl), Hinxton, United Kingdom; *http://www.ensembl.org*.].

### Western blot analysis

Cellular proteins were collected with SDS-lysis buffer containing protease inhibitor cocktail (Sigma Aldrich, St. Louis, MO, USA) and benzonase digested. Tissue was collected in RIPA buffer with PIC. Tissues were homogenated using a Dounce homogenizer. After centrifugation (14,000 rpm; 15 min; 4°C) and removal of eventual fat layers, protein concentrations were determined with the bicinchoninic acid protein assay kit (Pierce, Rockford, IL, USA). Primary antibodies used were anti-APMAP mouse monoclonal 46F raised against full-length human APMAP (epitope unknown; Abcam, Cambridge, MA, USA, or Novus Biologicals, Littleton, CO, USA), anti-GLUT4 (Merck Millipore, Billerica, MA, USA), anti-β-actin (Sigma-Aldrich), anti-His (GE Healthcare), anti-Flag (Sigma-Aldrich), anti-LOXL3 (Santa Cruz Biotechnology, Santa Cruz, CA, USA), anti-GAPDH (Cell Signaling Technology, Danvers, MA, USA), anti–α tubulin (Abcam), anti-phospho-Akt (Ser473) XP (Cell Signaling Technology), and anti-Akt (pan) (Cell Signaling Technology). Secondary antibody signals were visualized by ECL SuperSignal West Pico Chemiluminescence substrate (Pierce) or Amersham ECL prime substrate (GE Healthcare) using the G:Box detection system (Syngene, Frederick, MD, USA).

### Adipocyte and stromal vascular fraction isolation from eWAT, gonadal WAT, and stromal WAT

Fresh visceral and subcutaneous fat pads from mice were minced in 1 ml DMEM and digested in collagenase (1.5 U/ml in PBS; Sigma-Aldrich) and dispase II (2.4 U/ml; Sigma-Aldrich), with shaking for 30 min at 37°C. Digestion was stopped with DMEM containing FBS and cleared with a cell strainer. After centrifugation at 1000 *g* for 10 min at 20°C, the top white adipocyte fraction was collected and the supernatant discarded. The pellet was digested with 500 µl red blood cell lysis buffer (15.5 mM NH_4_Cl, 1 mM KHCO_3_, 10 µM EDTA, sterile filtered) for 5 min at room temperature. After adding 500 µl PBS with 0.5% bovine serum albumin, stromal vascular fraction (SVF) cells were pelleted. For cultivating SVF, the procedure was similar without red blood lysis, and cells were seeded directly onto 12-well plates. Preadipocytes from SVF were cultivated in DMEM/F-12 supplemented with 10% FBS and penicillin–streptomycin. The differentiation was similar to 3T3-L1 cells (17). Gonadal white adipocytes (gWACs) were differentiated in the presence of 1 µM rosiglitazone for the first 3 d.

### *In vivo* preadipocyte proliferation

Eight-wk-old male mice were treated with 0.8 mg/ml bromodeoxyuridine (BrdU) in drinking water and concomitantly put on HFD for 1 wk. Water was changed every 72 h. eWAT-derived SVF was collected as previously described. SVF cells were cultivated for 36 h, and anti-BrdU (1:100; Abcam) and Cy2 AffiniPure Goat Anti-Rat (1:200; Jackson ImmunoResearch Laboratories, West Grove, PA, USA) were used to visualize proliferating cells. Fixation, acid hydrolysis, and permeabilization were performed according to the manufacturer’s guidelines (anti-BrdU; Abcam). Slides were mounted with antifade mounting medium (Vector Laboratories, Burlingame, CA, USA) containing DAPI before microscopy.

### Microscopy

Microscopy was performed with a Leica SP5 confocal microscope with spectral detection (Leica Microsystems, Buffalo Grove, IL, USA) and a HCX IRAPO L ×25/0.95 NA water immersion objective.

Coherent anti-Stokes Raman scattering (CARS) microscopy was performed using a commercial setup consisting of a picosecond laser source and an optical parametric oscillator (picoEmerald; APE, Berlin, Germany) integrated into a Leica SP5 confocal microscope (Leica Microsystems) that enables 2-photon microscopy. DAPI was excited at 780 nm using the 2-photon laser source, and emission was detected using appropriate emission filters. Green fluorescent protein (GFP) was excited at 488 nm using an argon laser; emission was detected between 500 and 550 nm. DAPI and GFP signals were recorded sequentially. Single sections from 20 different positions within a sample were generated. The number of cells was determined by counting DAPI-stained nuclei. For automated registration of nuclei, DAPI-stained organelles were segmented for quantification using the Otsu method implemented in Fiji open-source software (*https://fiji.sc/*) (21). GFP signal in nuclei was determined visually on the basis of created DAPI and GFP overlay images. The average number of GFP positive nuclei per cell from 20 pictures per replicate was determined. Detection of the CARS signal of subcutaneous white adipocyte (sWAC) and epididymal white adipocyte lipid droplets was achieved using 650/210 nm emission filters and using a nondescanned detector in Epi mode. To detect neutral lipids/lipid droplets, the laser was tuned to 2845 cm^−1^, thus enabling imaging of CH_2_ symmetric stretching vibrations. *z* stacks were created at distances of 1 µm through the samples (field of view, 620 × 620 µm). Three-dimensional data were projected using the maximum-intensity projection method and Fiji software for representation. Image noise in acquired *z* stacks was reduced using 3-dimensional gaussian filtering (1/1/1 σ, *x*/*y*/*z*). *Z* stacks were projected using the maximum-intensity projection method. Lipid droplets were segmented using local thresholding described by Sauvola and Pietikäinen (22). The watershed method was applied to separate closely associated image objects. Segmentation results were manually refined if indicated. The mean area (µm^2^) of extracted image objects was calculated. Incomplete registered image objects at the border of the images were excluded from analysis. At least 6 *z* stacks covering one cell layer from arbitrary positions were acquired from each specimen. Inspection of 3-dimensional data was performed using Amira (FEI Co., Limeil-Brevannes, France). Image filtering, segmentation, and quantification of extracted image objects was performed by ImageJ open-source software (Image Processing and Analysis in Java; U.S. National Institutes of Health, Bethesda, MD, USA; *http://imagej.nih.gov/*).

### Human preadipocyte isolation and differentiation

Human subcutaneous AT was obtained from healthy individuals undergoing lipoaspiration. The detailed method has been described in Lindroos *et al*. (23). This study was approved by the Medical University of Vienna’s Ethics Committee and the General Hospital of Vienna (EK 1115/2010). All subjects provided written informed consent before taking part in the study.

### Mass spectrometry

Antibody-stained protein bands were excised from Western blot membranes, stripped, blocked, and digested with either modified trypsin (Promega) or chymotrypsin (Roche, Basel, Switzerland). Peptides were precipitated with acetone, dissolved in 0.3% formic acid and 5% acetonitrile, and separated by nano-HPLC (Dionex Ultimate 3000) equipped with a C18, 5 μm, 100 Å, 500 μm × 0.3 mm enrichment column and an Acclaim PepMap RSLC nanocolumn (C18, 2 μm, 100 Å, 50 cm × 0.075 mm) (all Thermo Fisher Scientific). Peptides were enriched and separated over a 180- or 300-min gradient. The samples were analyzed in an LTQ Orbitrap Velos Pro MS (Thermo Fisher Scientific) in positive ion mode by alternating full-scan mass spectrometry (MS) (*m/z* 300–2000) in the orbitrap (at 60,000 resolution) and tandem MS (MS/MS) by collision induced dissociation of the 10 most intense peaks in the ion trap with dynamic exclusion enabled, or in a maXis II ETD MS (Bruker, Bremen, Germany) operated with the captive source (capillary 1300 V, dry gas flow 3 L/min at 150°C, nanoBooster 0.2 bar) in positive mode by alternating full-scan MS (*m/z* 200–2000, scan rate 3.88 Hz) and MS/MS by collision-induced dissociation of the 17 most intense peaks with dynamic exclusion enabled (scan rate, 17 Hz). The Bruker MS data were analyzed by the data analysis software including internal recalibration with sodium formate clusters, and converted into MGF files by msConvert software (ProteoWizard; *http://proteowizard.sourceforge.net/*). For further data analysis together with Orbitrap RAW data, Proteome Discoverer 1.4 (Thermo Fisher Scientific) and Mascot 2.4 (Matrix Science, London, United Kingdom) were used. MS/MS data were analyzed by decoy database search containing the published proteome of *Mus musculus* (Swiss-Prot, *http://www.uniprot.org/*), the Apmap sequences (E1 and E2), and sequences of general lab contaminants. Detailed search criteria are as follows: enzyme, trypsin or chymotrypsin; maximum missed cleavage sides, 2; carbamidomethylation of cysteine as fixed modification; possible oxidation of methionine; precursor mass tolerance ±10 ppm; and product mass tolerance ±0.7 Da, 1% false discovery rate.

### 5′ Rapid amplification of cDNA ends

To obtain the 5′ end of the cDNA sequence, 5′ rapid amplification of cDNA ends (5′RACE) was performed using the Smarter RACE 5′/3′ kit (Clontech Laboratories, Mountain View, CA, USA) following the manufacturer’s protocol. High-quality total RNA from brown AT (BAT) of WT and ApmapE1-KO mice was used for the RACE kit. The following gene-specific primers (GSPs) were used for the 5′RACE PCR: E6 _GSP, 5′-GCCCTCAATGGGCGTCTCAGAGG-3′ and E8 _GSP, 5′-GCCAGAGCTGCTAGGCCGGATATTG-3′. The final RACE products were cloned into the pRACE vector provided with the kit, and 7 to 8 independent clones per construct were sequenced.

### Glucose tolerance test and insulin tolerance test

Mice were unfed before the glucose tolerance test (GTT) and the insulin tolerance test (ITT) for 6 or 4 h, respectively. In case of GTT, 1.5 g/kg glucose was injected intraperitoneally; 2 g/kg glucose was gavaged in oral GTT. For ITT, 0.5 U/kg insulin (or dose indicated in the figure caption) was intraperitoneally injected. Glucose was monitored with a glucose meter (Calla; Wellion, Marz, Austria) from tail venous blood.

### Plasma parameters

Plasma glucose levels were measured with a glucose meter. Commercially available kits were used to determine plasma triacylglycerol (TG) and free fatty acid contents. Plasma insulin levels were measured with the Mouse Ultrasensitive Insulin ELISA (Alpco Diagnostics, Salem, NH, USA) and the Adiponectin and Leptin with Mouse ELISA (Crystal Chem, Downers Grove, IL, USA) kits.

### Histology

Tissue samples were fixed in 4% buffered formaldehyde and embedded in paraffin. Sections were stained with hematoxylin and eosin or Trichrome according to standard protocols. Adipocyte size was assessed by NIS-Element software (Nikon Instruments, Tokyo, Japan). At least 3 areas per individual section per mouse fat pad were analyzed at ×200 magnification. Total adipocyte cell number in the depot was estimated as follows, as previously described (24): [*n* = *m* (depot mass g)/*P* (density of adipose 0.915 g/cm^3^) × volume (cm^3^)].

### Folch extraction

Liver neutral lipids were extracted from frozen tissue according to a standard extraction procedure (25). Thereafter, the chloroform phase was evaporated and the lipids resuspended in water containing 1% Triton X-100. TG content was assessed with a commercially available kit.

### Collagen content

Frozen eWAT was used for assessment of total collagen content using the commercially available Total Collagen Assay (QuickZyme Biosciences, Leiden, The Netherlands).

### Measurement of cellular reactive oxygen species production

Intracellular reactive oxygen species (ROS) production was determined using CellRox Deep Red Flow Cytometer Assay Kit (Thermo Fisher Scientific) according to the manufacturer’s protocol. Briefly, mature 3T3-L1 adipocytes overexpressing Apmap were incubated for 1 h at 37°C with 1 μM CellRox. Afterward, cells were collected and stained with 1 µM Sytox blue (Thermo Fisher Scientific) to exclude dead cells. After washing once with PBS, cells (5 × 10^5^) were analyzed by flow cytometry using an Attune NxT Acoustic Focusing Cytometer (Thermo Fisher Scientific).

### ^3^H-Deoxyglucose uptake

Mature 3T3-L1 cells stable overexpressing Apmap were starved in Krebs–Ringer buffer (KRB) (135 mM NaCl, 5 mM KCl, 1 mM MgSO_4_, 1 mM CaCl_2_, 20 mM Hepes, 0.4 mM KH_2_PO_4_, pH 7.4) supplemented with 2% bovine serum albumin (p/v) for 1.5 h. Afterward, cells were incubated in the absence or presence of insulin (200 ng/ml) in KRB for 30 min. Glucose uptake was determined using 1 mM d-glucose and 2-[^3^H]deoxyglucose (0.2 μCi/well) in KRB for 15 min. The assay was stopped by washing cells 5 times with ice-cold PBS. Cells were lysed with 0.5 M NaOH/0.1% SDS (p/v) by shaking for 4 h. Incorporated radioactivity was counted by liquid scintillation counting. Counts were normalized to protein concentrations measured by bicinchoninic acid.

### *De novo* lipogenesis

Incorporation of ^14^C-labeled glucose was measured as previously described (26)

### Body composition and indirect calorimetric measurements

Body composition was assessed with the miniSpec NMR Analyzer (Bruker Optics). For indirect calorimetric measurements, mice were individually housed in metabolic cages (LabMaster home cage system; TSE Systems, Bad Homburg, Germany) for 2–3 d (20–22°C, 13 h light/11 h dark cycle, lights on at 7:00 am). Mice were adapted to the metabolic cages for 2 d. Mice were provided with HFD and water *ad libitum*.

### Coimmunoprecipitation using Flag-M2-affinity gel

COS7 cells (ATCC) were cotransfected with plasmids pcDNA4/HismaxC containing full-length murine Apmap_E1 coding sequence and pFLAG-CMV-5.1 containing murine Loxl1 or Loxl3 coding sequences or pcDNA4/HismaxC containing Lox and pFLAG-CMV-5.1 containing Apmap_E1. Transfection was performed in 10 cm dishes with Metafectene Pro (Biontex, Munich, Germany) following the manufacturer’s guidelines. pcDNA4/HisMax/*lac*Z or empty pFLAG-CMV-5.1 served as a negative control. At 48 h after transfection, cell lysates were collected with coimmunoprecipitation buffer (50 mM Tris-HCl, pH 7.4–7.5; 300 mM NaCl; 1% Triton X-100) and cleared from cell debris *via* centrifugation, and protein content was measured. Lysate (1 mg) was used for pulldown with Anti-FlagM2 Affinity gel (Sigma-Aldrich) according to the manufacturer’s guidelines. After overnight incubation, beads were washed thoroughly, and affinity-bound proteins were eluted by boiling 2× SDS lysis buffer (100 mM Tris-HCl, pH 6.8; 10% glycerol; 2.5% SDS; 1× PIC). Immunoprecipitation products were immediately subjected to Western blot analysis.

### Statistical analysis

If not otherwise stated, results are expressed as means ± sd of at least 3 independent experiments, or results show 1 representative experiment out of 3. Statistical analysis was done on all available data. Statistical significance was determined by the 2-tailed Student’s *t* test or by 1-way ANOVA followed by a Bonferroni *post hoc* test. For statistical analysis, we used GraphPad Prism 7 software (GraphPad Software, La Jolla, CA, USA). Values of *P* ≤ 0.05 were considered significant; levels of significance are indicated in the figures.

## RESULTS

### Identification of an AT-specific APMAP isoform

**Figure 1.**
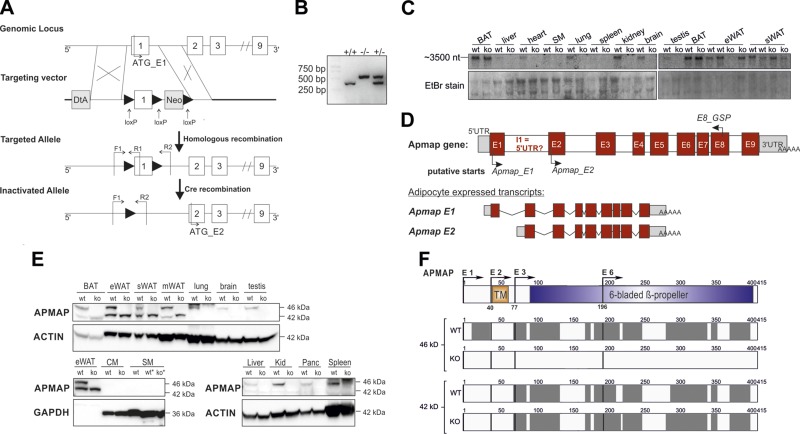
Targeted deletion of *Apmap* gene and identification of a second isoform. *A*) Scheme of targeting strategy. Exons are numbered and depicted as white boxes. In diphtheria toxin (DtA) containing targeting vector, loxP site was inserted upstream of exon 1 (containing ATG start codon), and loxP flanked neomycin cassette was inserted downstream of exon 1. Deletion of exon 1 was done in 2-step process (homologous recombination in ES cells and Cre recombination in mice). *B*) Identification of mouse genotypes by PCR with primers indicated in *A*. F1, forward primer; R1 and -2, reverse primers; +/+, WT mice; +/−, heterozygous ApmapE1-KO mice; −/−, homozygous ApmapE1-KO mice. *C*) Northern blot analysis of indicated tissues of WT and ApmapE1-KO mice. cDNA probe was located in 3′ region of *Apmap* gene. *D*) Scheme of *Apmap* primary transcript including GSPs used for 5′RACE PCR and transcripts detected by 5′RACE. E, exon; I, intron. *E*) Western blot analysis of indicated tissues of WT and ApmapE1-KO mice. CM, cardiac muscle; Kid, kidney; Panc, pancreas. One representative blot is shown of *n* ≥ 3 of AT; other tissues, *n* = 2–3. *F*) Top shows schematic organization of APMAP protein sequence. APMAP isoforms were identified by MS after detection by immunoblotting. Sequence coverage (gray boxes) and identified APMAP isoforms by MS of 46- and 42-kDa bands of WT and ApmapE1-KO eWAT, respectively, are shown. The 46-kDa band was not detected in ApmapE1-KO eWAT by immunoblotting, and APMAP was not identified by MS in excised area. Detected peptides are depicted in detail in Supplemental Fig. 2*C*. Data are representative for combined results of 2 independent experiments with *n* = 4. TM, transmembrane region.

To investigate the physiologic role of APMAP *in vivo,* exon 1 of the murine *Apmap* gene was disrupted by homologous recombination of the targeting vector with the *Apmap* gene locus in 129 HM-1 ES cells, followed by Cre-mediated recombination in mice (**Fig. 1*A***). ApmapE1-KO mice were generated by crossing heterozygous ApmapE1-KO mice, and genotypes were identified by PCR (Fig. 1*B*). To prove the deletion of *Apmap,* we performed Northern blot analysis with a cDNA probe located at the 3′ region of the *Apmap* coding sequence. According to the NCBI database, the full-length 46-kDa APMAP protein is encoded by a 2190-nt mRNA (NM_027977.2; GenBank). However, in control mice, we detected a transcript of about 3500 nt that was absent in liver, heart, lung, kidney, brain, and testis of ApmapE1-KO mice but that was still detectable in ATs [eWAT, subcutaneous WAT; stromal WAT (sWAT), brown AT (BAT)] (Fig. 1*C*). Further database research revealed the possible existence of 3 other transcripts (XM_006500198.2, XM_006500199.3, XM_017319266.1; GenBank). We refer to the various transcripts as full-length *Apmap*_E1 (start codon in exon 1), *Apmap*_E2 (putative start codon in exon 2, predicted mRNA 3004 nt, protein ∼42 kDa), *Apmap*_E3 (putative start codon in exon 3, predicted mRNA 1753 nt, protein ∼38 kDa) and *Apmap*_E6 (putative start codon in exon 6, predicted mRNA 1838 nt, protein ∼25 kDa). According to the NCBI database, these transcripts share a similar 3′ untranslated region (UTR) but differ in the 5′ UTR sequences. Using isoform-specific primers located in the unique 5′ UTR and the putative first transcribed exon of the suggested transcripts (Supplemental Fig. 1*A*), we confirmed the deletion of exon 1 in all tested tissues of our KO mouse model, whereas a primer pair that recognizes all transcripts (exon junction E7–8) and the putative variant E2 showed *Apmap* mRNA expression in eWAT, sWAT, and BAT (Supplemental Fig. 1*B*).

In addition to the real-time quantitative PCR (qPCR) analysis, we performed 5′RACE to identify transcripts with their according 5′ UTR that are produced from the *Apmap* gene. We used a GSP located in exon 6 or 8 (Fig. 1*D*) that should recognize all possible transcripts and mRNA from BAT because of its high *Apmap* expression (Supplemental Fig. 1*B*). With this method, we confirmed the expression of 2 transcripts (Apmap_E1 and Apmap_E2) in BAT of WT mice (Fig. 1*D* and Supplemental Fig. 1*D, E*), while ApmapE1-KO mice only expressed the Apmap_E2 transcript. The 5′ UTR of Apmap_E1 corresponded to the published version in Ensembl (27) (transcript ID MGP_C57BL6NJ_T0058630.1, Supplemental Fig. 1*C, D*), while the 5′ UTR of Apmap_E2 differs from the predicted sequence in the NCBI mRNA, which should be 1530 nt. Testing various clones, we identified 2 different 5′ UTRs for Apmap_E2 (Supplemental Fig. 1*E*); 33 nt that corresponded to a part of the published version of Apmap_E2 (XM_006500198.2, Supplemental Fig. 1*E**, *version 1) and 5 of 7 clones showed another 5′ UTR sequence that contained 75 nt corresponding to a different part of intron 1 (Supplemental Fig. 1*C**, *version 2). In line with the mRNA data, protein analysis confirmed that the full-length 46-kDa APMAP protein was undetectable in all tested tissues of ApmapE1-KO mice (Fig. 1*E*). However, a second protein band of about 42 kDa was detected in all AT depots (eWAT, sWAT, mesenteric and perirenal WATs, and BAT) and was not affected by the targeted deletion of exon 1 (Fig. 1*E*, quantification in Supplemental Fig. 2*A*). No evidence for Apmap E3 and E6 variants was found by qPCR, 5′RACE, or protein detection (Supplemental Fig. 2*B*). MS confirmed that the 42-kDa band detected by immunoblot analysis is a truncated version of APMAP (Fig. 1*F*, Supplemental Fig. 2*C*). Together, these data reveal a yet unknown murine, AT-specific APMAP isoform *in vivo*. This isoform is independently transcribed from exon 2, which was verified by 5′RACE.

### *Apmap* transcripts are differentially expressed during adipocyte differentiation

**Figure 2.**
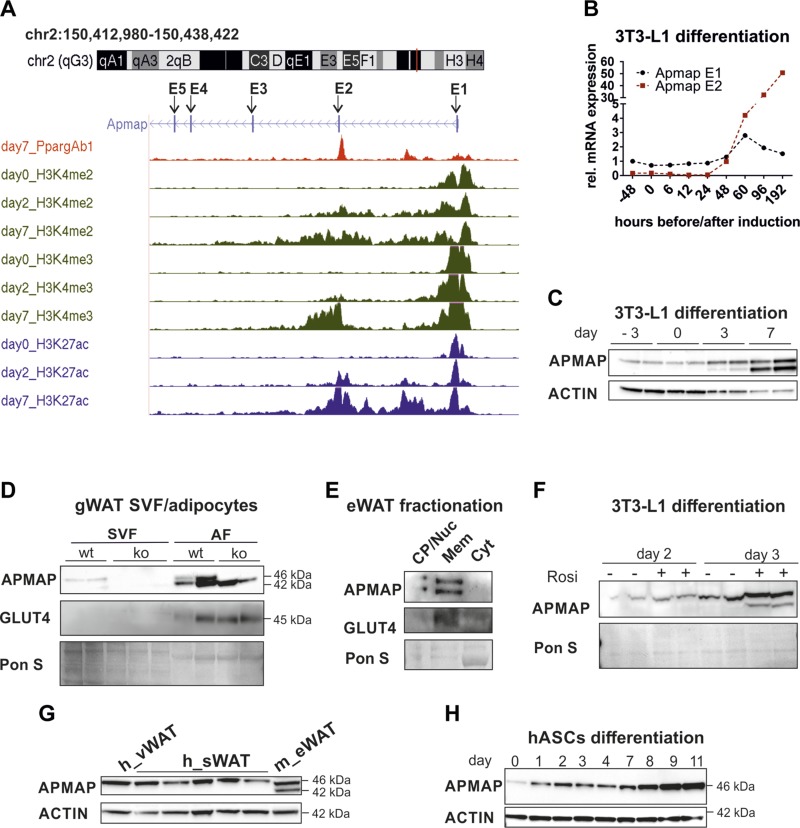
Apmap isoforms are differentially expressed in adipocytes. *A*) Custom tracks of ChIP-seq data from differentiating 3T3-L1 cells (28) uploaded to UCSC genome browser. Exons are depicted as E1–E5. *B*) mRNA and (*C*) protein expression of Apmap_E1 and E2 version during differentiation of 3T3-L1 cells. *D*) APMAP protein expression in SVF and adipocyte fraction (AF) of gonadal WAT (gWAT) of female ApmapE1-KO and WT controls. Each sample contains pool of gWAT fat pads of at least 2 mice. *E*) APMAP protein expression in fractionated eWAT. CP/Nuc, crude pellet/nucleus; Mem, total membranes; Cyt, cytosol. One representative of *n* = 3 is shown. *F*) APMAP protein expression in 3T3-L1 cells with and without rosiglitazone treatment (1 µM from d 0 until collection). *G*) APMAP protein expression in human visceral WAT (h_vWAT, *n* = 1) and subcutaneous WAT (h_sWAT, *n* = 5). One murine eWAT sample (m_eWAT) was applied. *H*) Protein expression of APMAP during differentiation of human adipose stromal cells (hASCs) into adipocytes; *n* = 1.

After identifying the AT-specific isoform Apmap_E2, we investigated whether *Apmap* transcripts are differentially regulated during differentiation of 3T3-L1 cells. Enrichments of certain methylations and acetylations of histone H3 indicate transcriptionally active sites. Thus, we examined the mouse *Apmap* locus in publicly available chromatin immunoprecipitation sequencing (ChIP-seq) data (28) and found that preadipocytes (d 0) contain active transcription start sites (enriched by methylated H3K4 and acetylated H3K27) around exon 1 (**Fig. 2*A***). No enrichment of H3K4me2/3 and H3K27ac signals around exon 2 could be detected in preadipocytes, but signals of both histone marks gradually increased starting with d 2 of differentiation, with robustly enriched signals on d 7 (data set accessible at NCBI Gene Expression Omnibus under accession number GSE20752, and depicted in Fig. 2*A* from ref. 28). We confirmed the active sites using the data set of Steger *et al.* (29). In their ChIP-seq experiments, they used antibodies against acetylation of lysine 9 in histone H3 (H3K9ac), another histone modification that strongly correlates with transcriptional active loci. Whereas preadipocytes contained an active H3K9ac mark around exon 1, a second active histone mark around exon 2 could only be detected in mature adipocytes [3T3-L1 d 10; data set accessible at NCBI Gene Expression Omnibus under accession number GSE21898 (29) or depicted in Supplemental Fig. 3*A*]. On the basis of these findings, we reassessed the expression of *Apmap* transcripts during the differentiation of 3T3-L1 adipocytes using transcript-specific primers. We observed the highest *Apmap_E1* mRNA expression 60 h after induction of differentiation, whereas *Apmap_E2* expression was undetectable before d 2 of differentiation and massively increased thereafter (Fig. 2*B*). Concomitantly, APMAP_E1 protein was already expressed in preconfluent 3T3-L1 cells, while APMAP_E2 protein was detectable only from d 3 onward and strongly increased until d 7 (Fig. 2*C*, quantification in Supplemental Fig. 3*C*). Further, APMAP_E1 was detectable in the SVF, whereas APMAP_E2 was expressed adipocyte-specific in murine visceral WAT (Fig. 2*D*). Both isoforms were located in the membrane fraction of murine eWAT (Fig. 2*E*). In our previous publication we showed that *ApmapE1* is a functional peroxisome proliferator-activated receptor γ (PPARγ) target (17). The PPARγ binding site is located between intron 1 and exon 2 around the putative transcription start site of *ApmapE2.* Accordingly, the PPARγ agonist rosiglitazone increased APMAP_E1 and APMAP_E2 (Fig. 2*F*). Interestingly, human adipocytes only express APMAP_E1, which is also up-regulated during differentiation of human adipose stromal cells into adipocytes (Fig. 2*G, H**;* quantification of *H* in Supplemental Fig. 3*C*). Also, no active histone marks could be found around exon 2 of the human *APMAP* gene (Supplemental Fig. 3*B*, adapted from ref. 28).

In summary, these data revealed that APMAP isoforms are differentially expressed during adipogenesis. In contrast to mice, human adipocytes only express the APMAP_E1 variant, and thus our KO mouse model lacks exactly the full-length APMAP variant that is conserved in humans.

### APMAP_E2 version is down-regulated on an HFD

**Figure 3.**
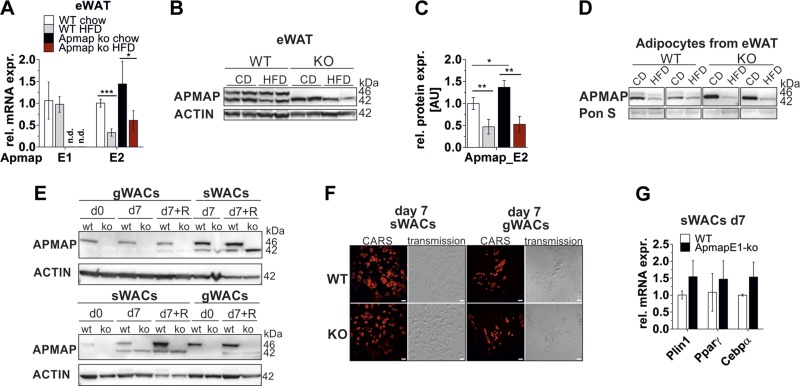
APMAP_E2 version is down-regulated on an HFD in eWAT. *A*, *B*) mRNA (*A*; *n* = 4–5) and protein (*B*) expression of APMAP_E2 is decreased after 22 wk of HFD. One representative blot of *n* = 4 is shown. CD, chow diet. *C*) Quantification of blot shown in *B* and further biologic replicates; *n* = 4. *D*) APMAP protein expression in adipocyte fraction of male chow and 20 wk HFD-fed ApmapE1-KO and WT mice. Two replicates are shown; *n* (chow) = 2–4; *n* (HFD) = 4. *E*) SVCs from female WT and ApmapE1-KO mice (WT *n* = 5, KO *n* = 4) were isolated and differentiated *in vitro*. APMAP protein expression in gWACs and in sWACs on d 0 and 7. Rosiglitazone (R, 1 µM) was added throughout differentiation to depicted samples. Two representative blots are shown. *F*) CARS and transmission pictures of WT and ApmapE1-KO sWACs and gWACs on d 7 of differentiation. One representative picture shown of 3 biologic replicates. Scale bars, 50 µm. *G*) mRNA expression of adipogenic genes in ApmapE1-KO sWACs on d 7; *n* = 3. *P ≤ 0.05, **P ≤ 0.01, ***P ≤ 0.001 (Student’s *t* test).

In our previous study, we found that *Apmap* is required for adipogenesis in 3T3-L1 cells and is deregulated in genetically obese mice (17). At that time we were unaware of the existence of the E1 and E2 isoforms, and the antibody we used also failed to detect the 42-kDa isoform. Thus, we were interested how the two isoforms are regulated in DIO and whether loss of one isoform affects fat cell development. APMAP_E2 mRNA and protein expression was significantly reduced in eWAT of HFD-fed WT and ApmapE1-KO mice (**Fig. 3*A, B****;* quantification in Fig. 3*C*). This diet-induced down-regulation of APMAP_E2 was even more evident when looking at the adipocyte fraction of eWAT (Fig. 3*D*). In liver and BAT, APMAP protein expression was not affected by HFD (Supplemental Fig. 4*A–D*). We isolated stromal vascular cells (SVCs) from eWAT and sWAT of WT and ApmapE1-KO mice and differentiated them *in vitro*. In undifferentiated SVCs, APMAP_E1 was present in WT cells, while neither isoform was detectable in ApmapE1-KO cells (Fig. 3*E*). As observed in 3T3-L1 cells, APMAP_E2 expression was up-regulated during SVC differentiation and further increased upon addition of rosiglitazone (Fig. 3*E**;* quantification in Supplemental Fig. 4*E*). The SVC differentiation capacity from WT and ApmapE1-KO mice was comparable, as shown by visualizing lipid droplets by CARS microscopy (Fig. 3*F*) and mRNA expression levels of adipogenic genes (*Pparγ, C/ebpα, Plin1*) in fully differentiated sWACs (Fig. 3*G*). *Apmap* silencing in 3T3-L1 cells impaired adipogenesis (17); however, the short hairpin RNA used for stable silencing in 3T3-L1 cells knocked down both isoforms during differentiation (Supplemental Fig. 4*F*, quantification Supplemental Fig. 4*G*). However, stable overexpression of APMAP_E1 in 3T3-L1 cells did not affect adipocyte differentiation, as shown by Oil Red O staining, glucose uptake or *de novo* lipogenesis, or ROS production in mature adipocytes (Supplemental Fig. 5*A–F*).

These data, taken together, indicate that APMAP_E2 is strongly reduced in DIO in WT and ApmapE1-KO mice and that adipocytes derived from ApmapE1-KO mice differentiated normally *in vitro*.

### APMAP_E1 deficiency in mice affects AT mass and adipocyte size on an HFD

**Figure 4.**
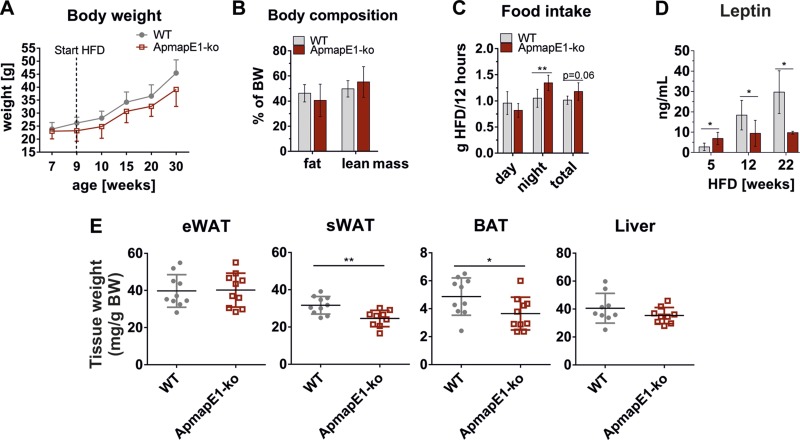
ApmapE1-KO affects AT mass and leptin levels on HFD. Male ApmapE1-KO and WT mice were put on HFD at age of 9 wk. *A*) Weight was measured every wk. Here, 5-wk intervals are shown; *n* = 10. *B*) Body composition was measured in Bruker Minispec NMR after 14 wk of HFD; *n* = 6. *C*) Food intake was measured in metabolic cages after 10 wk of HFD. Average food intake per 12-h light or dark period is shown; *n* = 6. *D*) Plasma leptin levels after 5, 12, and 22 wk of HFD. Blood was drawn in refed (5- and 12-wk time point) and fed *ad libitum* state (22 wk time point); *n* = 4–6. *E*) Tissue was excised after 22 wk of HFD, and wet weight was measured and calculated relative to mouse body weight; *n* = 10. *P ≤ 0.05, **P ≤ 0.01 (Student’s *t* test).

**Figure 5.**
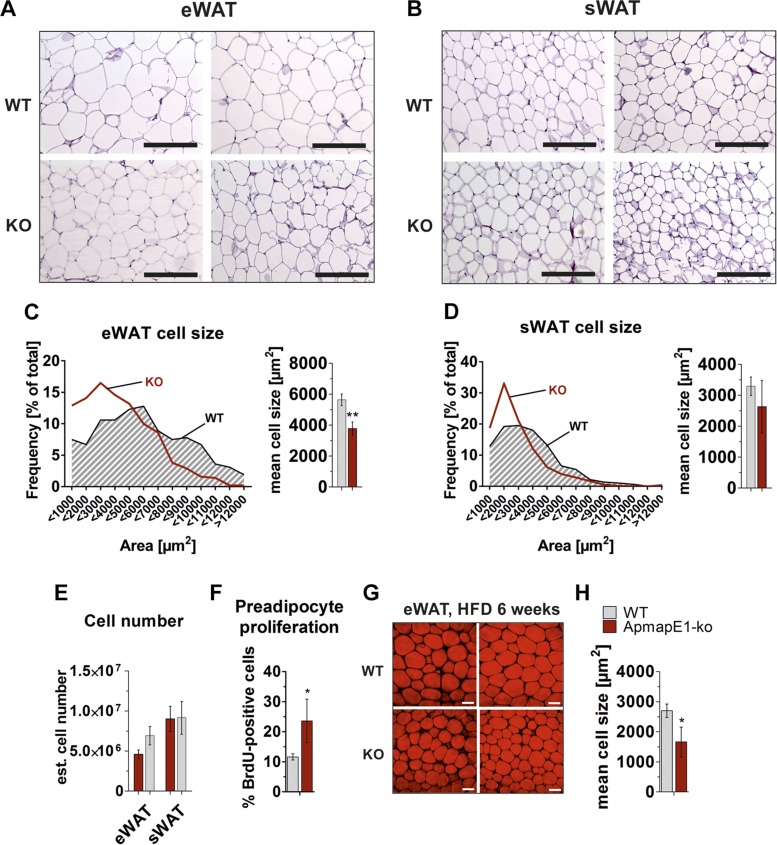
ApmapE1-KO mice have reduced adipocyte size on HFD. *A, B*) Representative images of hematoxylin and eosin–stained sections of eWAT (*A*) and sWAT (*B*) from WT and ApmapE1-KO mice after 22 wk of HFD. Scale bars, 200 µm. *C, D*) Corresponding eWAT (*C*) and sWAT (*D*) adipocyte size was analyzed by NIS-Element software. At least 3 different areas per fat pad section of each mouse were quantified, and distribution of adipocyte areas was calculated; *n* = 3–4. *E*) Estimated cell number calculated from hematoxylin and eosin–stained eWAT and sWAT sections (24). ApmapE1-KO and WT mice were fed HFD for 22 wk; *n* = 3–4. *F*) Preadipocytes were isolated from eWAT of male ApmapE1-KO and WT mice after BrdU treatment (0.8 mg/ml in drinking water) and HFD feeding for 1 wk. Cells were seeded on coverslips and stained for DAPI and BrdU. BrdU-positive cells were calculated by Fiji software (21). *G*) CARS microscopy was used to visualize neutral lipids in eWAT of WT and ApmapE1-KO mice after 6 wk of HFD; *n* = 3. Scale bars, 50 µm. *H*) Quantification of adipocyte size of *F*. *P ≤ 0.05, **P ≤ 0.01 (Student’s *t* test).

On a chow diet, ApmapE1-KO mice and WT controls show comparable body weight, tissue weight, and glucose and insulin tolerance (Supplemental Fig. 6*A–F*). On an HFD, no significant changes in weight gain and body composition between ApmapE1-KO mice and WT controls were observed (**Fig. 4*A, B***), although food intake was significantly increased in ApmapE1-KO mice during the dark phase (Fig. 4*C*). Accordingly, leptin levels remained low in ApmapE1-KO mice, whereas they increased in WT mice (Fig. 4*D*). However, femur length was not different between both genotypes (WT = 15 ± 0.4 mm; ApmapE1-KO = 14.8 ± 0.3 mm; *n* = 4). ApmapE1-KO mice had significantly reduced sWAT and BAT weight, while eWAT, muscle, and liver weights were unchanged compared to controls (Fig. 4*E* and Supplemental Fig. 7*A, B*). Also, liver TG content did not differ between WT and ApmapE1-KO (Supplemental Fig. 7*C*). Histologic examination and CARS microscopy of fat depots revealed that adipocyte size was significantly decreased in eWAT of ApmapE1-KO mice already after 6 wk on an HFD (**Fig. 5*F, G***) and still after 22 wk of HFD feeding (Fig. 5*A, C*). However, we only detected a trend to an elevated number of fat cells in this depot (Fig. 5*E*). To test whether this trend to an elevated fat cell number was due to increased proliferation, mice were fed an HFD and BrdU-supplemented drinking water for 1 wk. Importantly, we assessed a significantly increased preadipocyte proliferation in ApmapE1-KO eWAT already within 1 wk of HFD (Fig. 5*F*). Also, a trend to smaller adipocytes was observed in sWAT (Fig. 5*B, D*), while the cell number was unchanged (Fig. 5*E*). BAT morphology did not differ between ApmapE1-KO and WT mice (Supplemental Fig. 8*A*). Because BAT size was decreased on an HFD in ApmapE1-KO mice, we investigated whether BAT activity was changed in these mice. However, cold tolerance as well as *Ucp1* and *Cidea* mRNA expression were unaffected, whereas *Prdm16* mRNA expression was reduced in ApmapE1-KO mice (Supplemental Fig. 8*B–D*). These data revealed that ApmapE1-KO is beneficial for the white AT phenotype when fed an HFD.

### Apmap_E1 deletion promotes a metabolically healthy phenotype on an HFD

**Figure 6.**
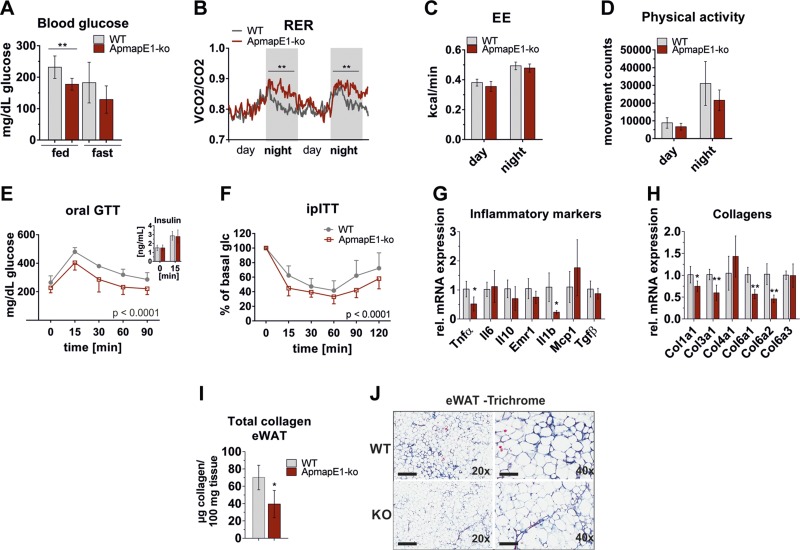
ApmapE1-KO show improved metabolic phenotype on an HFD. *A*) Blood glucose of WT and ApmapE1-KO mice measured in fed *ad libitum* and overnight-unfed (“fast”) state after 12 wk of HFD; *n* = 6. *B*) Respiratory exchange ratio (RER) was measured after 2 d of adaption period for further 2.5 d in metabolic cages; *n* = 6. *C*) Energy expenditure (EE) was calculated from *V*co_2_ and *V*o_2_ measured in metabolic cages; *n* = 6. *D*) Physical activity was assessed in metabolic cages; *n* = 6. *E*) Glucose tolerance (1.5 g glucose/kg body weight) and insulin tolerance tests (0.5 U insulin/kg body weight; *F*) were performed in male WT and ApmapE1-KO mice after 12 and 8 wk of HFD, respectively; *n* = 4 for GTT, *n* = 10 for ITT. Statistics calculated with 2-way ANOVA with Bonferroni *post hoc* test using GraphPad Prism software. *F*) Inlet plasma insulin levels before and after glucose bolus. *G, H*) mRNA expression of inflammatory markers (*G*) and collagens (*H*) in eWAT of WT and ApmapE1-KO mice (22 wk of HFD); *n* ≥ 4. *I*) Total collagen in eWAT was measured after 22 wk of HFD; *n* = 8–10. *J*) Trichrome-stained sections of eWAT from ApmapE1-KO and WT mice after 20 wk of HFD. Maximal fibrosis/collagen staining of each group is shown. One representative replicate is shown. Scale bars: 500 µm (left), 200 µm (right); *n* = 4. *P ≤ 0.05, **P ≤ 0.01 (Student’s *t* test).

Because ApmapE1-KO mice had smaller adipocytes in eWAT and less sWAT, we asked whether metabolic parameters are influenced in these mice when fed an obesogenic diet. ApmapE1-KO mice gained weight similar to WT controls and revealed a couple of comparable plasma parameters such as plasma free fatty acid (Supplemental Fig. 7*D*), TG (Supplemental Fig. 7*E*), adiponectin (Supplemental Fig. 7*F*), and insulin levels after a glucose bolus (**Fig. 6*E***, inset). However, ApmapE1-KO mice also exhibited a number of features of metabolically healthy obesity. They showed decreased blood glucose levels in the fed *ad libitum* state (Fig. 6*A*). Corroborating these data, we observed enhanced glucose oxidation in ApmapE1-KO mice during the night, reflected by an increased respiratory exchange ratio (Fig. 6*B*), while energy expenditure and physical activity were unchanged (Fig. 6*C, D*). Moreover, ApmapE1-KO mice showed improved glucose tolerance (Fig. 6*E*) and increased insulin sensitivity compared to WT mice (Fig. 6*F*). To investigate whether skeletal muscle (SM) contributes to improved glucose metabolism, we examined AKT phosphorylation in SM and compared it to AT and liver. Insulin signaling was unchanged in SM, liver, and eWAT, while we saw a trend to increased AKT phosphorylation in sWAT (Supplemental Fig. 7*J*). Further, we measured mitochondrial marker gene expression in SM but found these genes unchanged or even reduced (Supplemental Fig. 7*I*). While marker gene expression for ROS and endoplasmic reticulum stress was unchanged in eWAT and sWAT (Supplemental Fig. 7*G, H*), the expression of inflammatory markers that are involved in the progression of insulin resistance like Tnf-α and Il-1β was reduced in eWAT of HFD-fed ApmapE1-KO animals (Fig. 6*G*). Interestingly, profibrotic collagens (Col1a1, -3a1, -6a1 and -6a2; Fig. 6*H*) and total tissue collagen content (Fig. 6*I*) were significantly decreased in eWAT of ApmapE1-KO mice on HFD. Supporting the biochemical data, we observed decreased Trichrome staining for collagen in eWAT of HFD-fed ApmapE1-KO mice compared to WT mice (Fig. 6*J*); however, there was considerable variation within the groups. Together, these results indicate decreased fibrotic potential in ApmapE1-KO mice and show that deletion of Apmap_E1 is beneficial for mice receiving an HFD.

### APMAP interacts with ECM proteins LOXL1 and LOXL3 and affects their expression level

**Figure 7.**
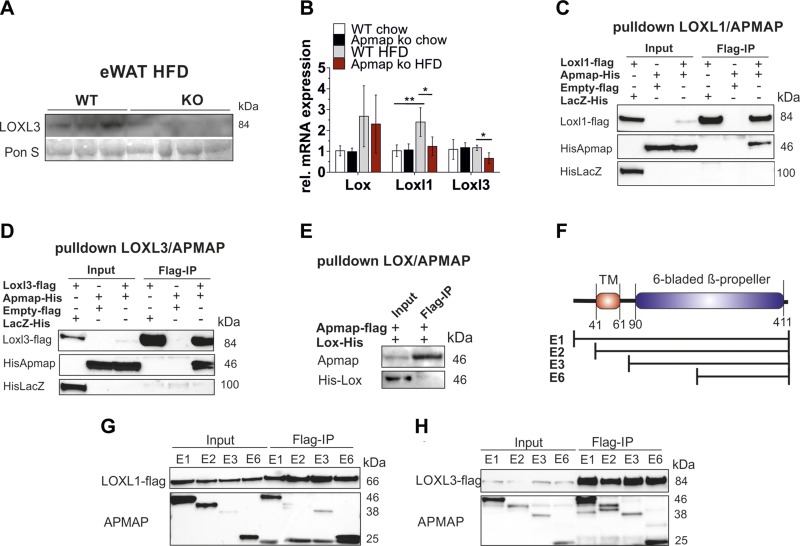
APMAP interacts with LOXL1 and 3 and influences their expression level. *A*) Protein expression of LOXL3 in eWAT of HFD-fed ApmapE1-KO and WT mice; *n* = 3–4. *B*) mRNA expression of Lox, Loxl1, and Loxl3 in eWAT; *n* ≥ 4. *C–E*) Interaction of APMAP_E1 with LOXL1 (*C*), LOXL3 (*D*), and LOX (*E*) in COS7 cells. Pulldown was performed with flag beads. COS7 cells were transfected with Loxl1/-3-flag or Apmap-flag vectors and His-Apmap or His-Lox vector. Empty pCMV 5.1 flag and His-*LacZ* served as controls. One representative pulldown of *n* ≥ 3 is shown. *F*) Scheme of APMAP full-length and truncated protein variants. TM, transmembrane region. *G, H*) Flag beads pulldown with LOXL1-flag (*G*) and LOXL3-flag (*H*) and APMAP_E1 and truncated variants (E2, -3, -6) in COS7 cells. *P ≤ 0.05, **P ≤ 0.01 (Student’s *t* test).

We found decreased profibrotic collagen in ApmapE1-KO mice, and a high-throughput yeast 2-hybrid analysis revealed that human APMAP interacts with LOXL3 (30). Thus, we reasoned that Apmap expression could affect this collagen cross-linking protein and is thereby involved in ECM regulation. Our data show that the robust expression of LOXL3 in eWAT is strongly decreased in HFD-fed ApmapE1-KO mice on the protein and mRNA level (**Fig. 7*A, B***). Also, the mRNA expression of the isoenzyme Loxl1, which is strongly increased on an HFD, was diminished in eWAT of ApmapE1-KO mice, while the expression of Lox was not influenced by *Apmap_E1* disruption (Fig. 7*B*). Interestingly, coimmunoprecipitations with LOX, LOXL1, and LOXL3 revealed that APMAP interacts with LOXL1 (Fig. 7*C*) and LOXL3 (Fig. 7*D*), but not with LOX (Fig. 7*E*). Further, we assume that the region responsible for coimmunoprecipitation with LOXL1 and -3 is located within the C-terminal region of APMAP (scheme depicted in Fig. 7*F*) because all truncated APMAP variants share this region and interact with LOXL1 (Fig. 7*G*) and LOXL3 (Fig. 7*H*) when overexpressed in COS7 cells.

Because lysyl oxidases are of critical importance for ECM composition by cross-linking collagen and elastin, these data suggest that APMAP, by interacting with LOXL1 and LOXL3 protein, affects ECM remodeling/composition.

## DISCUSSION

Identifying novel candidates that modulate the metabolic phenotype in obesity are of great interest to develop new therapeutic tools. In this study, we characterized APMAP regarding its role in AT expansion during the development of DIO. Our previous report suggested an important role of APMAP in adipocyte development *in vitro,* but no physiologic data were available at that time.

### Apmap isoform expression is tissue and species specific

We targeted exon 1 harboring the transcription start site of the gene. At that time it was not known that several APMAP isoforms may exist, and the antibody used against APMAP failed to detect alternative isoforms (17). During the characterization of the KO mouse, we identified a novel APMAP isoform (variant E2) that is exclusively expressed in adipocytes. APMAP_E2 misses the intracellular N-terminal domain that might have regulatory properties, as it contains a putative phosphorylation site at threonine 19 (Uniprot Q9D7N9). Using 5′RACE, we identified 2 5′ UTRs of Apmap_E2 in murine BAT; however, the complete sequence remains unknown. We assume that intron 1 contains another promoter region that transcribes *Apmap* in a tissue-specific manner. This is realistic because deletion of exon 1 did not abolish the expression of the transcript in ATs and enriched methylation at lysine 4 of histone H3 (H3K4me3) around exon 2 is an indicator for promotor regions (28). Moreover, alternative transcription using an alternative transcription start is a common occurrence in mammalian genomes (31, 32). Use of alternative promoters enables diversification of transcriptional regulation within a single locus and thereby plays a significant role in the control of gene expression in various cell lineages, tissue types, and developmental stages (31). In humans, the situation seems to be different. It was reported that the human APMAP protein appears in 2 splice forms (50/52 kDa and 30/32 kDa). The truncated isoform might lack exons 3 to 5. Both of these putative splice variants contain the N-terminal cytosolic domain (33). However, it has not been proven experimentally that the truncated protein version is APMAP. Using the available monoclonal antibody that was raised against human full-length APMAP (epitope unknown), we only detected one isoform in human SVCs and AT lysates corresponding to the murine APMAP_E1 variant.

Alternative transcribed isoforms are often associated with distinct functional or regulatory properties (31). APMAP_E1 is expressed ubiquitously and is already present in preadipocytes, while APMAP_E2 only appears after the initial differentiation phase, when the conversion into mature adipocytes takes place. Additionally, histone modifications indicating active loci appear around exon 2 in a differentiation-dependent manner. The adipocyte specificity of Apmap_E2 is also reflected by its preferential expression in the adipocyte fraction of murine AT. HFD feeding decreases the expression of many genes that are normally increased during adipocyte differentiation (34). We show that APMAP_E2 isoform is also robustly diminished on an HFD. However, we can only speculate why APMAP_E2 is reduced in eWAT while it is unchanged in BAT. APMAP as a PPARγ target gene might be differentially regulated in WAT and BAT. Although WAT and BAT share many pathways, especially during adipogenesis, it has been shown that these adipose depots have distinct regulatory circuits (35–37) and that on an HFD, but also caloric restriction, tissues strongly differ in the extent and nature of their transcriptomic response (38, 39). Therefore, a differential regulation of APMAP in eWAT and BAT is quite feasible. But as APMAP_E2 is down-regulated to the same extent in eWAT of WT and ApmapE1-KO mice on an HFD, we assume that the phenotype we see in ApmapE1-KO mice is due to the complete knockout of the APMAP_E1 protein. We are aware that only a complete-knockout mouse could answer some remaining questions. Because HFD further reduces total APMAP expression, ApmapE1-KO mice under this condition best resemble the whole-body knockout mouse. In addition, our data suggest that ApmapE1-KO leads to a “healthy obesity” phenotype by affecting ECM remodeling. Alterations in ECM remodeling have been shown to correlate with the adverse obesity-associated effects (9, 14, 40). This might partially explain why we only see a beneficial phenotype in ApmapE1-KO mice on an HFD but not on a chow diet.

### Apmap regulates AT expansion and hallmarks of metabolic health during obesity

Here we provide the first evidence that APMAP is involved in regulating adipose composition and consequently metabolic health in obesity. ApmapE1-KO mice showed multiple beneficial effects on an HFD. ApmapE1-KO mice are protected against diet-induced insulin resistance and show overall enhanced glucose utilization on an HFD. Obesity progression is accompanied by AT expansion, a process that is either accomplished by hyperplasia or hypertrophy (2). It has been suggested that adipocyte hyperplasia in visceral and subcutaneous fat correlates with a healthy metabolic phenotype in patients with obesity (41, 42). In contrast, large hypertrophic adipocytes lose their flexibility to deal with the nutritional overload and therefore become dysfunctional and favor insulin resistance (42). Data from ApmapE1-KO mice propose hyperplasia in eWAT, although AT mass did not change. Moreover, ApmapE1-KO mice have decreased expression of profibrotic collagens, accompanied by decreased TNF-α expression in eWAT. Human visceral AT is prone to inflammation in obesity because of enhanced immune cell content and increased proinflammatory cytokine expression like TNF-α that promotes insulin resistance in peripheral tissues. Chronic low-grade AT inflammation thereby represents a central trigger for the development of insulin resistance (43, 44). Leptin levels increase under HFD conditions, directly correlating with increased leptin resistance (45), while they remain low in ApmapE1-KO mice. The sWAT phenotype of ApmapE1-KO mice was less pronounced, but their reduced sWAT mass might be explained by trends to reduced cell size, although cell number is unchanged. Additionally, it is well studied that AT depots react differently to DIO (46) and that depot-specific adipocyte differentiation is influenced by their extracellular environment (47). In summary, the global absence of Apmap_E1 in mice on HFD ameliorates obesity-related metabolic disturbances.

### APMAP orchestrates ECM composition in AT during obesity

The adipose ECM affects the functional integrity of mature adipocytes (48). Recent studies have shown that alterations in adipose ECM remodeling on an HFD correlate with tissue fibrosis, inflammation, and insulin resistance (9, 14, 40). A role for LOX has already been described in AT (9, 14, 16). It has been shown that LOX expression is highly increased in humans and rats in DIO and that inhibition of LOX by a specific inhibitor ameliorates collagen content and metabolic profile in DIO in rats (14). Furthermore, LOX contributes to the commitment of preadipocytes to become mature adipocytes *via* bone morphogenic protein 2– and -4–mediated pathway (49, 50). However, little is known about Loxl1 and 3 in AT (14). Here we show that in addition to Lox, Loxl1 and -3 are highly expressed in ATs (*C_t_* values in eWAT range from Lox ∼27, Loxl1 ∼24, and Loxl3 ∼28). As described for Lox (14), Loxl1 expression is highly increased in DIO, and this increase is completely blunted in ApmapE1-KO mice. Additionally, LOXL3 expression was strongly decreased in eWAT of ApmapE1-KO mice, while Lox expression was not influenced by Apmap disruption in DIO. Interestingly, APMAP protein solely interacts with LOXL1 and -3, while it does not interact with LOX. Thus, it can be speculated that the interaction of APMAP with LOXL1 or LOXL3 promotes their stabilization. Also, APMAP might be involved in the transport of LOXL1 and -3 to the membrane, or it could act as a tethering protein at the cell surface. We assume this because studies from Mosser *et al.* (51) showed that APMAP is involved in the degradation of amyloid β and in the lysosomal pathway. They further suggested that APMAP might be bound to endosomal particles. However, in their study, silencing of Apmap led to increased amyloid β production, whereas in our case LOXL3 protein is decreased.

Although the detailed consequences of the interaction of APMAP with LOXL proteins need to be investigated in future, our data imply a critical function for APMAP in ECM remodeling and AT expansion.

## Supplementary Material

Supplemental Data

## Abbreviations

APMAP: adipocyte plasma membrane–associated protein
ApmapE1/2: Apmap exon 1/2
AT: adipose tissue
BAT: brown adipose tissue
BrdU: bromodeoxyuridine
CARS: coherent anti-Stokes Raman scattering
ChIP-seq: chromatin immunoprecipitation sequencing
DIO: diet-induced obesity
ECM: extracellular matrix
ES: embryonic stem
eWAT: epididymal white adipose tissue
FBS: fetal bovine serum
GFP: green fluorescent protein
GSP: gene-specific primer
GTT: glucose tolerance test
gWAC: gonadal white adipocyte
HFD: high-fat diet
ITT: insulin tolerance test
KO: knockout
KRB: Krebs–Ringer buffer
LOX: lysyl oxidase; LOXL1–4, lysyl oxidase-like 1–4
MS: mass spectrometry
MS/MS: tandem mass spectrometry
NCBI: National Center for Biotechnology Information
PPARγ: peroxisome proliferator-activated receptor γ
qPCR: quantitative PCR
RACE: rapid amplification of cDNA ends
ROS: reactive oxygen species
SM: skeletal muscle
SVC: stromal vascular cell
SVF: stromal vascular fraction
sWAC: subcutaneous white adipocyte
sWAT: stromal white adipose tissue
TG: triacylglycerol
UTR: untranslated region
WAT: white adipose tissue
WT: wild type
