# Supplementary material for: APMAP interacts with lysyl oxidase–like proteins, and disruption of Apmap leads to beneficial visceral adipose tissue expansion
Source: FASEB J. 2017 May 30;31(9):4088–103. doi: 10.1096/fj.201601337R (PMC5566180; doi:10.1096/fj.201601337R)
Supplement: Supplemental Data [file supp_31_9_4088__index.html]

APMAP interacts with lysyl oxidase–like proteins, and disruption of Apmap leads to beneficial visceral adipose tissue expansion — APMAP interacts with lysyl oxidase–like proteins, and disruption of Apmap leads to beneficial visceral adipose tissue expansion — Supplemental Data 

# APMAP interacts with lysyl oxidase–like proteins, and disruption of *Apmap* leads to beneficial visceral adipose tissue expansion

## Supplemental Data

- Supplemental Data
