# Supplementary material for: APMAP interacts with lysyl oxidase–like proteins, and disruption of Apmap leads to beneficial visceral adipose tissue expansion
Source: FASEB J. 2017 May 30;31(9):4088–103. doi: 10.1096/fj.201601337R (PMC5566180; doi:10.1096/fj.201601337R)
Supplement: Supplemental Data [file supp_fj.201601337R_Supplemental_Figures.pdf]

Supplemental data to manuscript „APMAP interacts with lysyl oxidase-like protein and disruption of Apmap leads to beneficial visceral adipose tissue expansion – Pessentheiner et al.

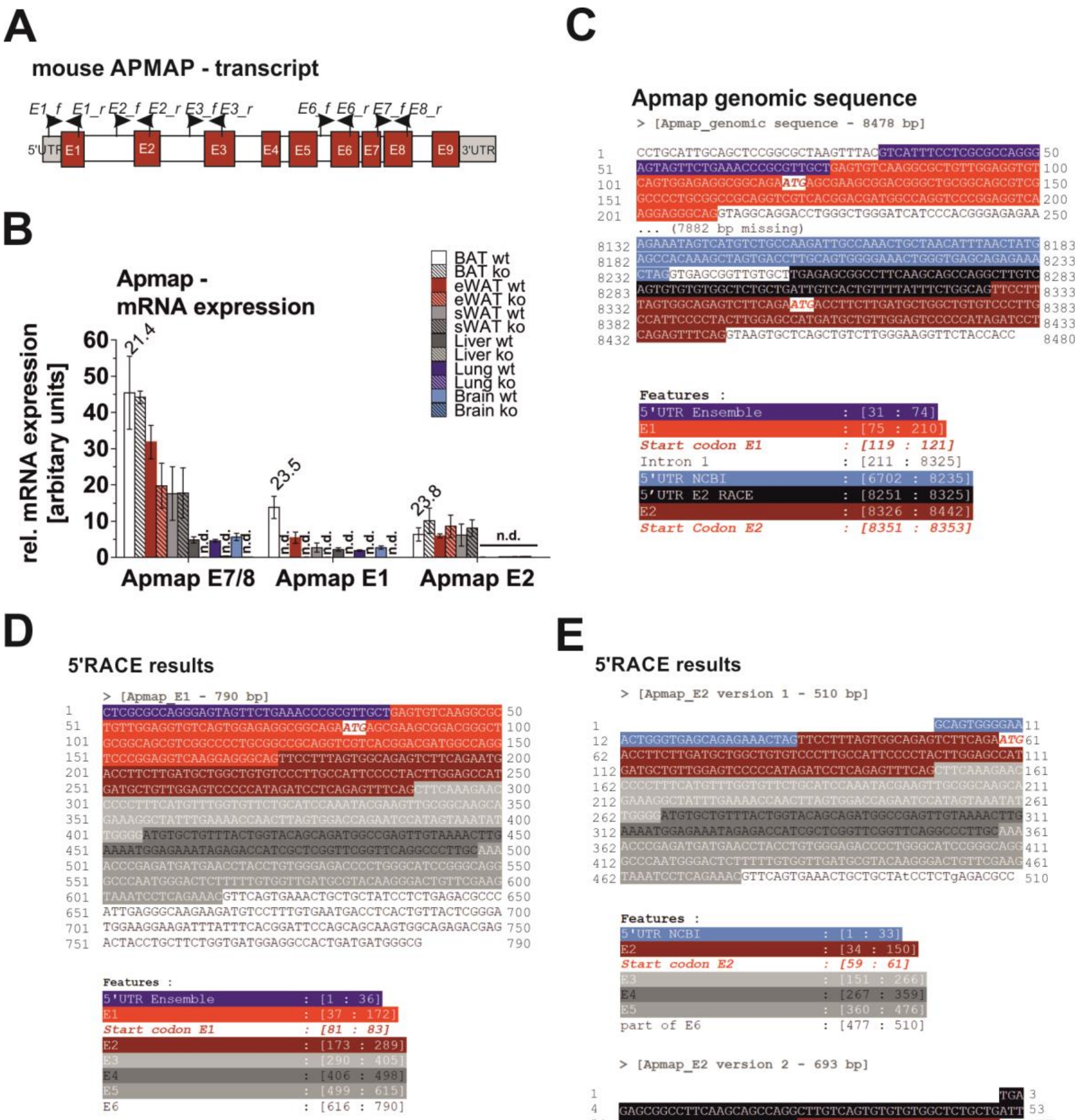

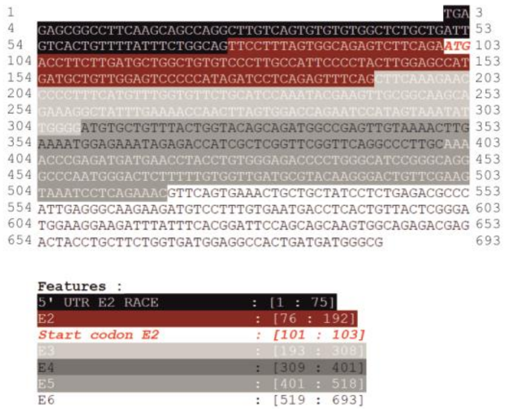

Figure S1: (A) Scheme of Apmap primary transcript including exons (E) and introns (white boxes). The primer pairs used for qPCRs are indicated with arrows and written in italics. (B) qPCR analysis of several mouse tissues (ApmapE1-ko and wt controls) with primers located in exon/exon junction 7/8 that recognize all Apmap versions and primers specific for *Apmap_E1* or *Apmap_E2*. Respective Ct-values are indicated above the bars. n ≥ 4. (C) Genomic sequence of Apmap gene sequence including exon 1 and 2 and their respective upstream and downstream regions. (D) & (E) The longest, sequenced 5’UTR regions of Apmap_E1 (D) and two versions of Apmap_E2 (E) obtained by 5’ RACE PCR of BAT wt cDNA are shown.

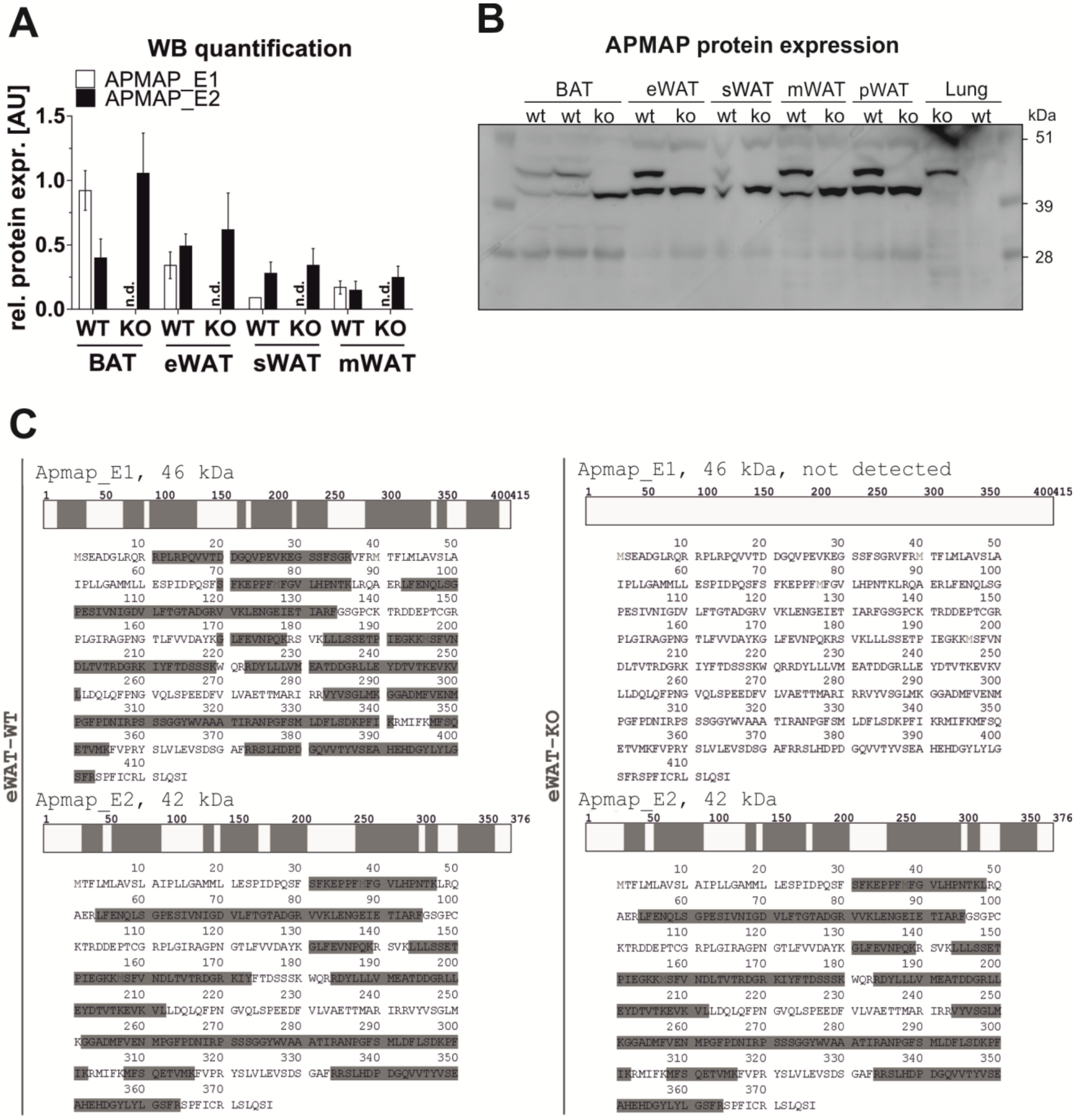

Figure S2: (A) Western blot quantification of APMAP_E1 and E2 relative to ACTIN from blot shown in Fig. 1E. Additional blots were used for quantification. n = 3 (except sWAT wt n = 2). (B) Uncropped Western blot from adipose tissues and lung. One representative blot is shown. (C) Mass spectrometry of APMAP isoforms. Sequence coverage (grey boxes) and identified APMAP isoforms of mass spectrometry of 46 kD and 42 kD bands of wt and ko eWAT, respectively, after detection by immunoblotting using an APMAP antibody are shown. No 46 kD band was detected in ko by immunoblotting and no APMAP was identified by mass spectrometry of the excised area. Detected sequences are depicted in detail.

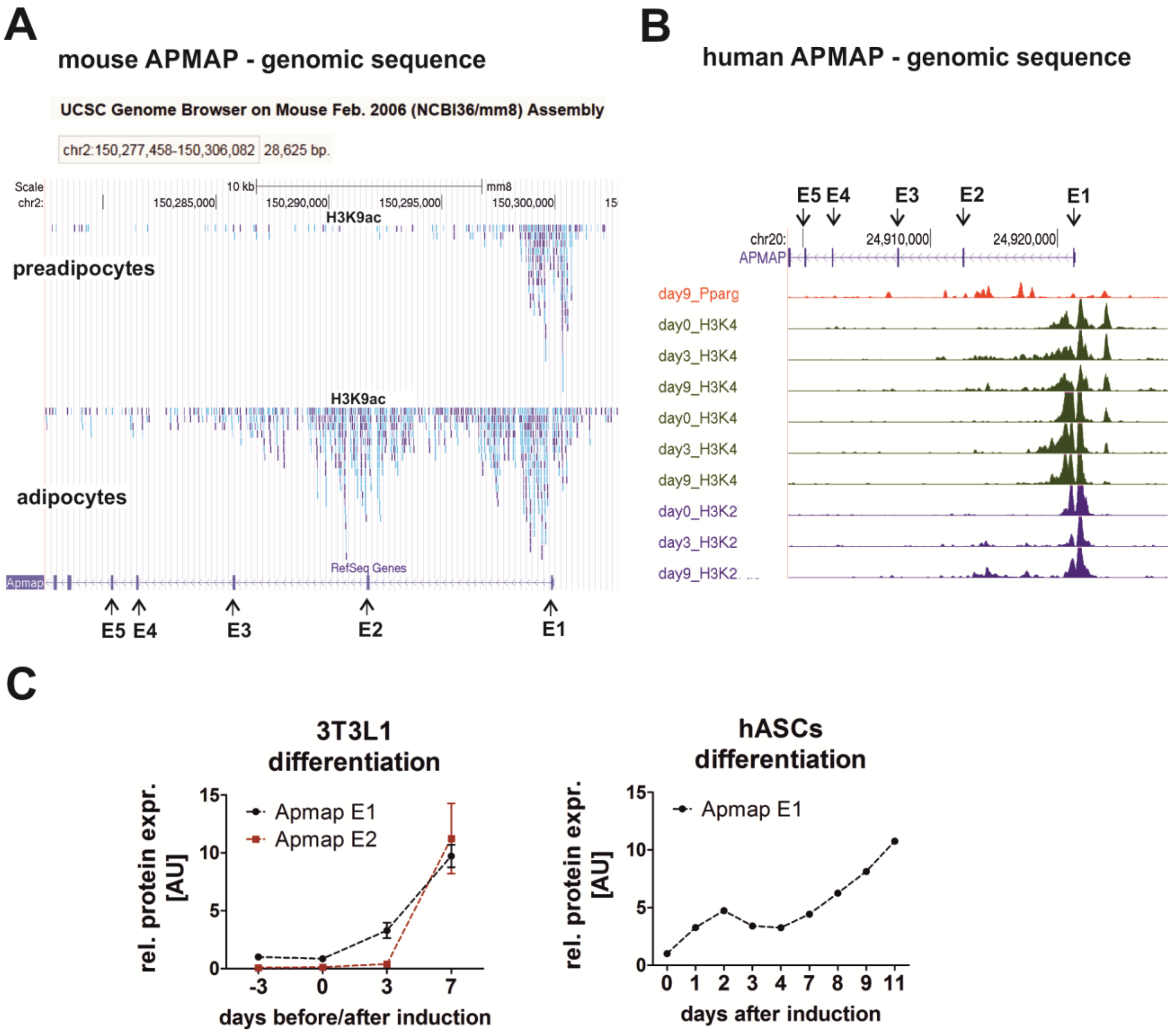

Figure S3: (A) Custom tracks of ChIP data from preadipocytes and adipocytes (3T3-L1 day 10) from (1) uploaded into the UCSC genome browser. (B) Custom tracks of ChIP data from differentiating human adipocyte derived stromal vascular cells from (2) uploaded into the UCSC genome browser. (C) Western blot quantification of APMAP_E1 and E2 relative to ACTIN from blots shown in Fig. 2C and 2H.

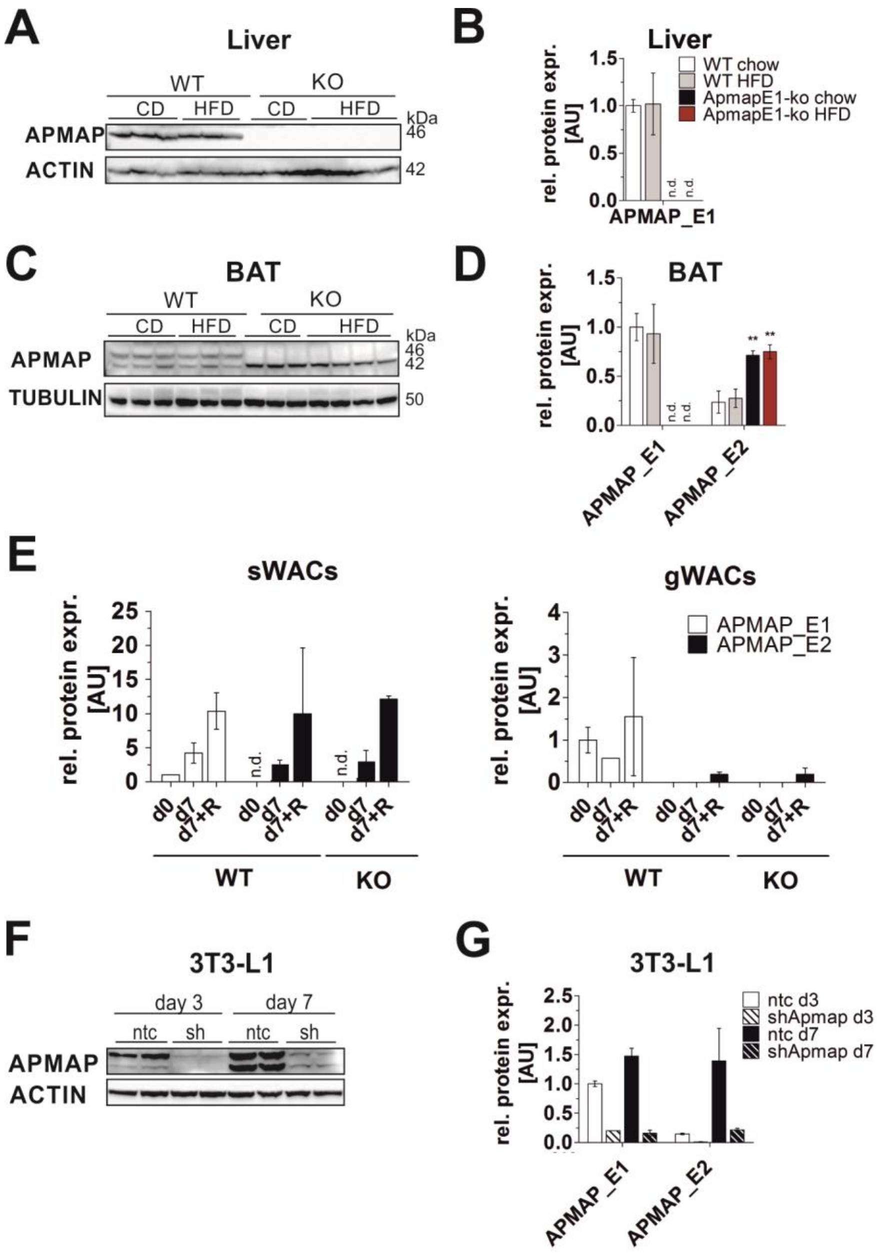

Figure S4: (A)-(D) Protein expression of APMAP and the according quantification of APMAP_E1 and E2 relative to ACTIN in liver (A, B) and relative to Tubulin in BAT (C, D) of chow and HFD fed wt and ApmapE1-ko mice. n = 3-4. (E) Western blot quantification of APMAP_E1 and E2 relative to ACTIN from blots shown in Fig. 3E. (F)-(G) Protein expression of APMAP and the according quantification of APMAP_E1 and E2 relative to ACTIN in shRNA Apmap stable-silenced 3T3-L1 cells on day 3 and 7 of differentiation. One representative blot with n = 2 is shown. ntc, non-targeting control.

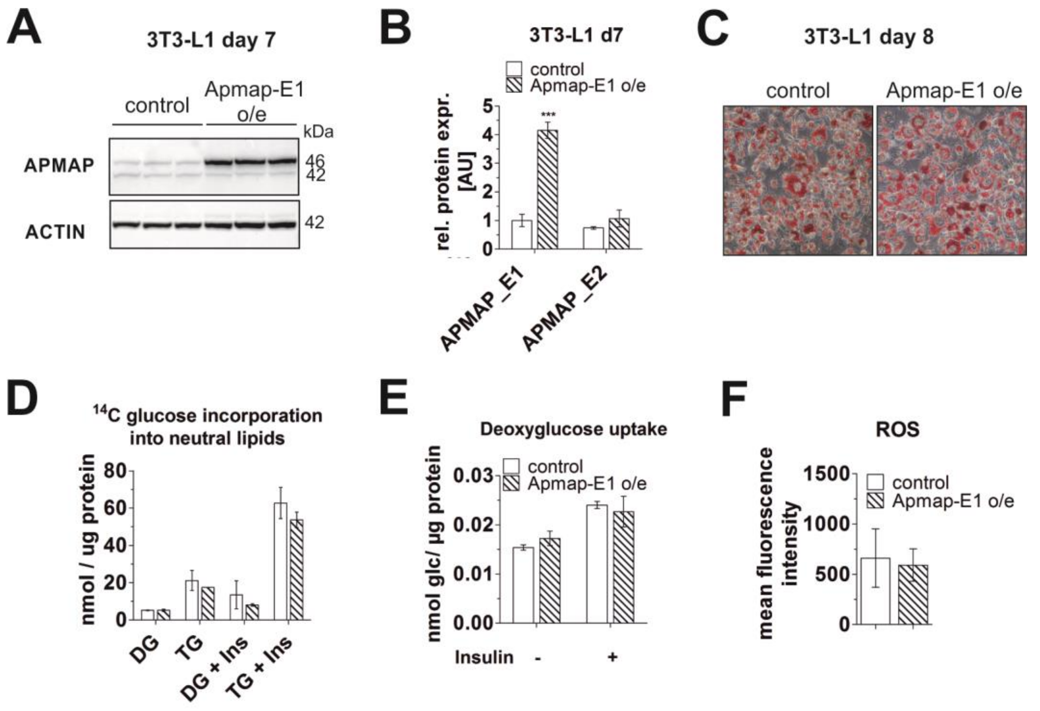

Figure S5: (A)-(B) Protein expression of APMAP (A) and the according quantification of APMAP_E1 and E2 relative to ACTIN (B) in control and ApmapE1 overexpressing 3T3-L1 cells at day 7 of differentiation. n = 3. (C) Oil red O staining of control and ApmapE1 overexpressing 3T3-L1 cells at day 8. Shown is one representative replicate out of three. (D) ^{14}C-Glucose incorporation into neutral lipids of control and ApmapE1 overexpressing 3T3-L1 cells at day 7 in basal state and upon insulin stimulation. n = 3. (E) ^{3}H-Deoxyglucose uptake of control and ApmapE1 overexpressing 3T3-L1 cells at day 7 in basal state and upon insulin stimulation. n = 2. (F) ROS measurement in control and ApmapE1 overexpressing 3T3-L1 cells at day 7 using flow cytometry. n = 3.

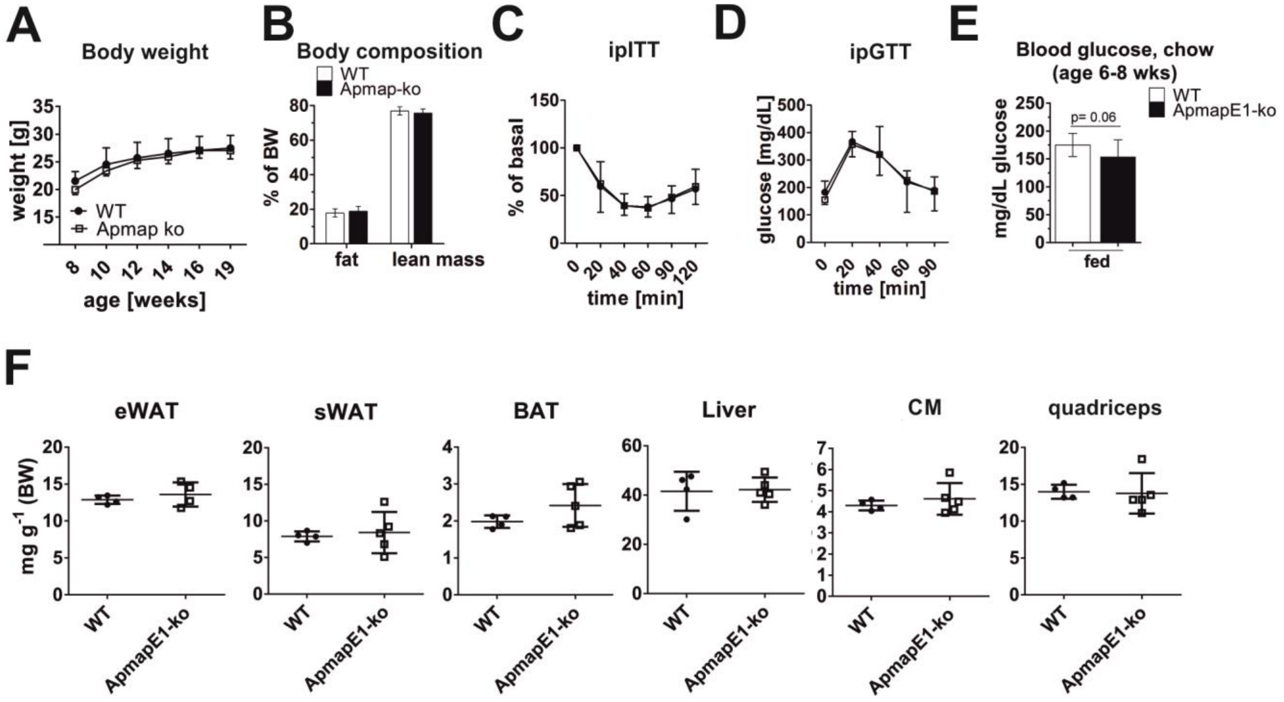

Figure S6: All data were generated from mice on chow diet. (A) Weight of ApmapE1-ko mice and wt littermates. n = 4. (B) Body composition measured in Bruker Minispec NMR at the age of 8 weeks. n ≥ 10. (C) Insulin tolerance (0.4 U insulin/kg BW) and (D) glucose tolerance (1.5 g glucose/kg BW) tests were performed in male WT and ApmapE1-ko mice at the age of 16 and 20 weeks, respectively. n ≥ 4. (E) Blood glucose was measured at the age of 6 to 8 weeks. n ≥ 10. (F) At the age of 34-41 weeks, tissue was excised and wet weight was measured and calculated relative to mouse body weight. CM, cardiac muscle. n ≥ 4.

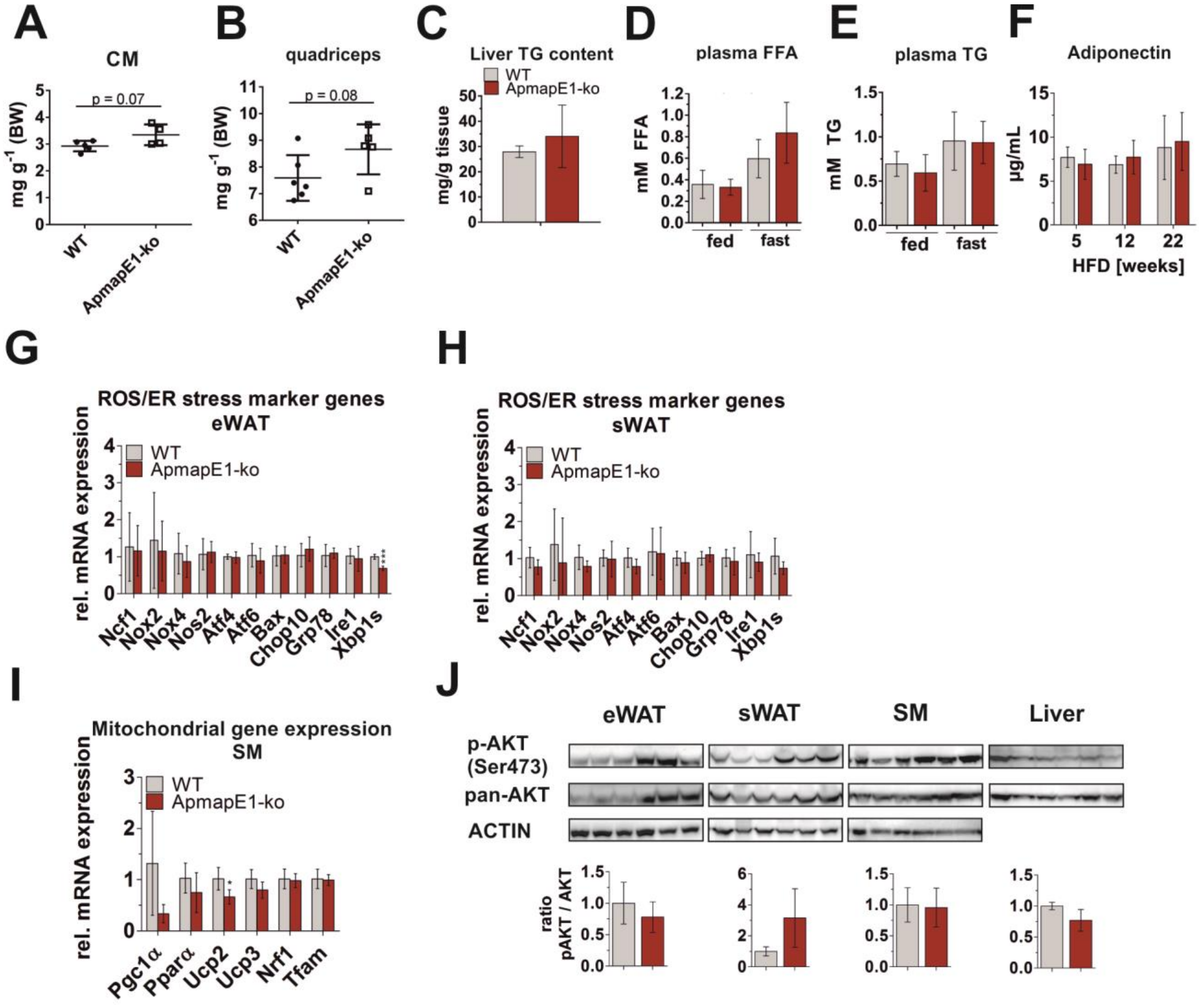

Figure S7: All data were generated from mice on HFD diet. (A) & (B) Tissue was excised and wet weight was measured and calculated relative to mouse body weight. CM, cardiac muscle. n = 5-6. (C) Liver triglyceride (TG) content was measured after Folch extraction. wt n = 5; ko n = 4. Plasma (D) free fatty acids (FFA) and (E) triacylglycerol (TG) measured from fed ad libitum or overnight fasted ApmapE1-ko and wt mice. n ≥ 10. (F) Plasma adiponectin levels after 5, 12 and 22 weeks of HFD. Blood was drawn in refed (5 and 12 week time point) and fed ad libitum state (22 week time point). n = 4-6. (G) & (H) ROS and ER stress marker gene expression in eWAT (G) and sWAT (H) of wt and ApmapE1-ko mice. n ≥ 4. (I) Mitochondrial marker gene expression in SM of wt and ApmapE1-ko mice. n ≥ 4. (J) Protein expression of pAKT and panAKT and the according quantification of the pAKT/AKT ratio in liver, SM, eWAT, and sWAT of wt and ApmapE1-ko mice 20 min after insulin injection (0.8 U/kg). n = 3. (t-test * p ≤ 0.05; *** p ≤ 0.001)

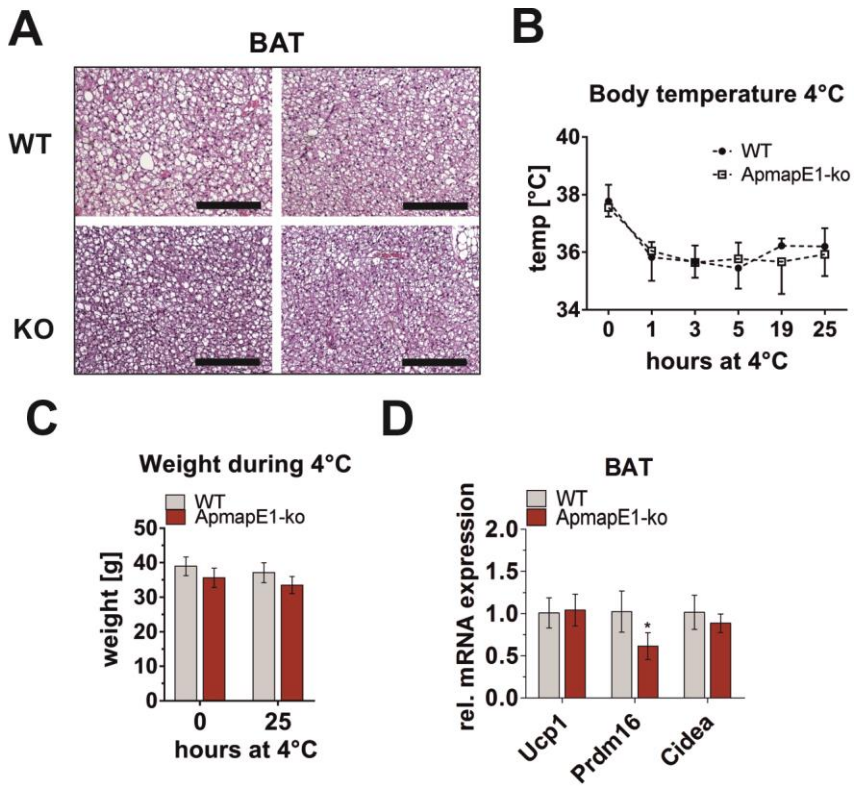

Figure S8: (A) Representative images of H&E stained sections of BAT from male ApmapE1-ko and wt littermates after 22 weeks on HFD. Scale bar = 200 µm. (B) Body temperature of ApmapE1-ko and wt mice during 25 hours acute cold-challenge. (C) Body weight of ApmapE1-ko and wt mice before and after 25 hours acute cold-challenge. For (B) and (C) animals were on HFD for 9 weeks. n = 4. (D) mRNA expression of brown marker genes of 22-week HFD fed ApmapE1-ko and wt mice. n = 4 (t-test * $p \leq 0.05$)

1. Steger, D. J., Grant, G. R., Schupp, M., Tomaru, T., Lefterova, M. I., Schug, J., Manduchi, E., Stoeckert, C. J., and Lazar, M. A. (2010) Propagation of adipogenic signals through an epigenomic transition state. *Genes Dev.* **24**, 1035–1044

2. Mikkelsen, T. S., Xu, Z., Zhang, X., Wang, L., Gimble, J. M., Lander, E. S., and Rosen, E. D. (2010) Comparative epigenomic analysis of murine and human adipogenesis. *Cell* **143**, 156–169
